# 25th Conference of GP2A

**DOI:** 10.3390/ph10040097

**Published:** 2017-12-14

**Authors:** Michael D. Threadgill, Susan E. Matthews, Francesca Giuntini

**Affiliations:** 1 Drug & Target Discovery, Department of Pharmacy & Pharmacology, University of Bath, Bath BA2 7AY, UK; 2 School of Pharmacy, University of East Anglia, Norwich NR4 7TJ, UK; susan.matthews@uea.ac.uk; 3 School of Pharmacy and Biomolecular Sciences, James Parsons Building, Liverpool John Moores University, Liverpool L3 3AF, UK; f.giuntini@ljmu.ac.uk

**Keywords:** medicinal chemistry, drug design, natural product, structure-based, prodrug

## Abstract

The 25th Conference of GP2A was held on 31 August and 1 September 2017 in Liverpool, UK, with the aim of exchange of ideas and experience, particularly amongst young medicinal chemists. Topics included bioactive compounds from plants and lichens, and design and development of drugs. Abstracts of invited lectures, proffered oral presentations, flash presentations and posters presented during the meeting are collected in this report.

## 1. Aims and Scope

The GP2A (Groupement des Pharmacochimistes de l’Arc Atlantique) is a group of academic medicinal chemists working in universities and research institutes near the western coast of Europe (the “Atlantic Arc”). It was founded in 1992 with the aim of bringing together researchers in this area for exchange of ideas and experience. Historically, it has included members from France, Spain, Portugal, Ireland and the United Kingdom. It has recently expanded both geographically and in terms of research field, now including researchers from physical and pharmaceutical chemistry to molecular pharmacology.

The aim of the annual conference is exchange of ideas and experience, particularly amongst young researchers. This is achieved in two ways, focussed conferences and facilitation of short exchange visits. Each year, we hold a meeting designed to bring together not only laboratory heads (Principal Investigators) but also postdocs and postgraduate research students (PhD students). At these meetings, everyone has the opportunity to present and discuss their latest research through invited lectures, oral communications and posters. In 2017, the conference was held in Liverpool John Moores University, Liverpool, UK. The major research topics that were presented included bioactive compounds from plants and lichens, synthetic methodology and design and development of drugs to treat cancer, diabetes, inflammation, infections and other diseases. These presentations were in the forms of lectures by invited experts, proffered oral presentations, posters with flash oral presentations and a large number of posters from young scientists, PhD students and postdoctoral researchers.

## 2. Invited Lectures

### 2.1. A Natural Products Story: Two Examples in the Alkaloids and Siderophore Series (IL1)

DuvalOlivierUFR Santé, Department of Pharmacy, 16 boulevard Daviers, University of Angers, SONAS, SFR 4207 QUASAV, 49100 Angers, France; olivier.duval@univ-angers.fr

Natural Products (NPs) traditionally have played an important role in drug discovery and were the basis of a lot of medicinal chemistry projects. We will discuss on the last decade example projects which were completed in our laboratory in two different fields, alkaloids heterocyclic compounds and siderophore series (Clere, N., et al. *Molecules* **2017**, *22*, 627; Clere, N., et al. *Carcinogenesis* **2011**, *32*, 286–295; Ouchani, F., et al. *Investig. New Drugs* **2014**, *33*, 75–85; Ouchani, F., et al. *Anal. Cell. Pathol.* **2012**, *35*, 267–284; Aissaoui, R., et al. *J. Org. Chem*. **2012**, *77*, 718–724; Bertrand, S., et al. *Mini-Rev. Med. Chem*. **2013**, *13*, 1311–1326; Bertrand, S., et al. *Tetrahedron Lett*. **2010**, *51*, 2119–2122).

### 2.2. Using Synthetic Chemistry to Solve Biological Problems—From Daffodils to Drugs (IL2)

AzubuikeDarlingtonCiupaAlexTunbridgeGemma A.GriffithsNatalie J.JuddKatie E.CaggianoLorenzo*Department of Pharmacy & Pharmacology, University of Bath, Claverton Down, Bath BA2 7AY, UK*Correspondence: l.caggiano@bath.ac.uk

Our research interests are the in the rational design and efficient synthesis of novel biologically active compounds, with applications in cancer and other diseases. We are also interested in the application of organic chemistry to biological systems and have developed a novel method for cell aggregation with potential applications in tissue engineering or as 3D tumour models. The presentation will focus on our latest results in these research areas. 

The synthesis of a maltol-derived hydrazide will be described which, once attached to a cell surface, induces rapid multicellular aggregation selectively in the presence of Fe^3+^ ions, which has potential applications in bioprocessing and regenerative medicine (Ciupa, A., et al. *Chem. Commun*. **2013**, *49*, 10148–10150). The rational design, synthesis and antiproliferative activity of various biologically active compounds (Ciupa, A., et al. *MedChemComm* **2011**, *2*, 1011–1015; Tunbridge, G.A., et al. *MedChemComm* **2013**, *4*, 1452–1456; Ciupa, A., et al. *MedChemComm* **2013**, *4*, 956–961), and our synthetic approach to various analogues of natural products (i.e., narciclasine), will also be described. Narciclasine is a natural product obtained from the bulbs of various *Amaryllidaceae* plants, such as daffodils, and is of particular interest due to its potent anti-tumour properties and potential for the treatment of primary brain tumours. The talk will present our latest efforts towards the efficient synthesis of novel narciclasine analogues based on synthetic methodology developed by our group (Judd, K.E., et al. *Synthesis* **2009**, *16*, 2809–2817).


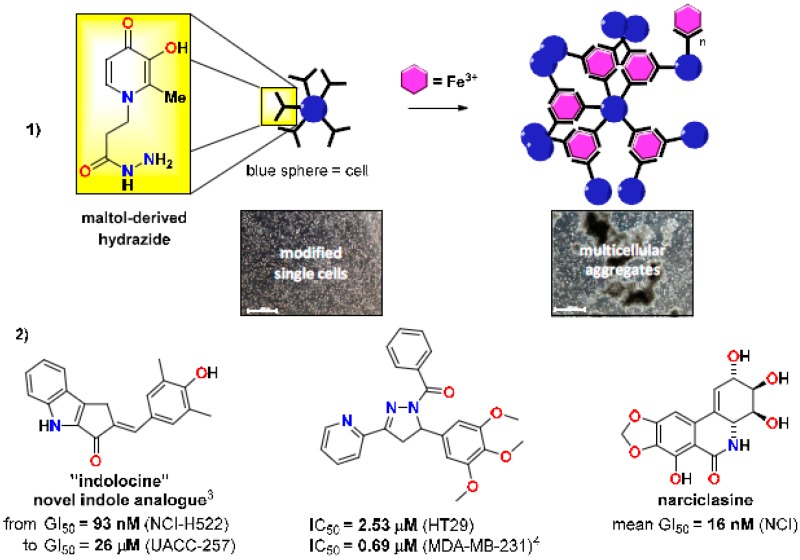


### 2.3. Cytotoxic Compounds from the Genus Centaurea L. (Asteraceae) (IL3)

SarkerSatyajit D.*NaharLutfunMedicinal Chemistry and Natural Products Research Group, School of Pharmacy and Biomolecular Sciences, Faculty of Science, Liverpool John Moores University, James Parsons Building, Byrom Street, Liverpool L3 3AF, UK*Correspondence: s.sarker@ljmu.ac.uk


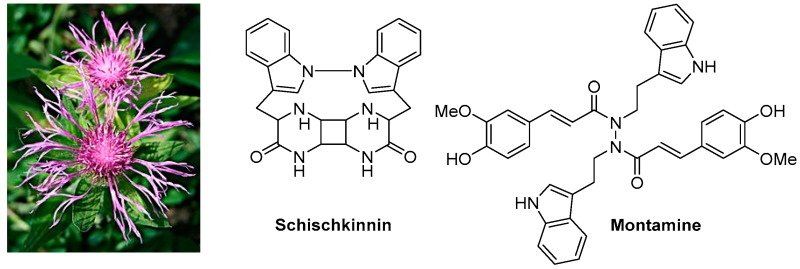


The Centaurea (family: *Asteraceae* alt. *Compositae*), commonly known as ‘centaury’, ‘centory’, ‘starthistles’ or ‘knapweeds’, comprises ca. 500 species of herbaceous thistles and thistle-like flowering plants, most of which are native to the Old World but also cultivated elsewhere. Many of these species are well known for their medicinal properties and are used as traditional medicines for the treatment of a number of ailments including diabetes, diarrhoea, hypertension, malaria, rheumatism, tumours and cancers. Several secondary metabolites, belonging to the classes of alkaloids (mainly tryptamine/serotonin derivatives), lignans (predominantly dibenzylbutyrolactone type), flavonoids, sesquiterpenes (generally lactones) and simple phenolics, with cytotoxic activities (potential anticancer properties), have been reported from different species of this genus. The presentation will give an overview of our on-going research on the chemistry of the genus *Centaurea*, and studies on cytotoxicity of various extracts and fractions as well as isolated secondary metabolites from the *Centaurea* species (Shoeb, M., et al. *Tetrahedron* **2006**, *62*, 11172–11177; Shoeb, M., et al. *Phytochemistry* **2006**, *67*, 2370–2375; Shoeb, M., et al. *ARS Pharm.* **2006**, *47*, 315–322; Shoeb, M., et al. *Tetrahedron* **2005**, *61*, 9001–9006; Shoeb, M., et al. *DARU* **2004**, *12*, 87–93; Sarker, S.D., et al. *Phytochemistry* **2001**, *57*, 1273–1276; Sarker, S.D., et al. *Nat. Prod. Lett*. **1997**, *9*, 189–199), mainly from the Turkish flora, against cancer cell lines.

### 2.4. Development of DNA-PK Inhibitors for Cancer Therapy (IL4)

CanoCelineNorthern Institute for Cancer Research, School of Chemistry, Newcastle University, Newcastle Upon Tyne NE1 7RU, UK; celine.cano@ncl.ac.uk

The cellular response to DNA double-strand break (DSB) formation is an essential component of normal cell survival, following exposure to DNA-damaging chemicals and ionising radiation (Hoeijmakers, J.H.J. *Nature* **2001**, *411*, 366–374). The serine/threonine kinase DNA-dependent protein kinase (DNA-PK) is a member of the phosphatidylinositol (PI) 3-kinase-related kinase (PIKK) family of enzymes, and plays an important role in DNA DSB repair via the non-homologous end-joining (NHEJ) pathway (Jackson, S.P. *Carcinogenesis* **2002**, *23*, 687–696). DNA-PK inhibitors may, therefore, be useful as agents to improve the activity of radio- and chemo-therapy in the treatment of cancer (Boulton, S., et al. *Carcinogenesis* **1996**, *17*, 2285–2290). Identification of the lead benzo[h]chromen-4-one DNA-PK inhibitor NU7026 (IC_50_ = 0.23 μM), guided the subsequent development of the potent and selective ATP-competitive chromenone NU7441 (DNA-PK IC_50_ = 30 nM) (Hardcastle, I.R., et al. *J. Med. Chem*. **2005**, *48*, 7829–7846). Although proof-of-principle studies with NU7441 confirmed promising activity in vitro as a chemo- and radio-potentiator in a range of human tumour cell lines (Zhao, Y., et al. *Cancer Res*. **2006**, *66*, 5354–5362), further biological studies with NU7441 were hampered by sub-optimal pharmaceutical properties. In collaboration with AstraZeneca Pharmaceuticals, structure-activity relationship studies were conducted in conjunction with homology modelling. This approach predicted several positions on the pendant dibenzothiophen-4-yl substituent of NU7441 as tolerant to substitution, without detriment to DNA-PK inhibitory activity. I will describe the rational design and syntheses of analogues that optimised the physicochemical and pharmacokinetic properties of NU7441. These studies resulted in the identification of compounds that combined potent DNA-PK inhibition, selectivity and excellent aqueous solubility (KU-0060648; DNA-PK IC_50_ = 8.6 nM). The discovery and further development of KU-0060648 and analogues will be described, including in vivo efficacy and combination studies (Cano, C., et al. *J. Med. Chem*. **2013**, *56*, 6386–6401; Cano, C., et al. *J. Med. Chem*. **2010**, *53*, 8498–8507; Clapham, K.M., et al. *Org. Biomol. Chem*. **2012**, *10*, 6747–6757).


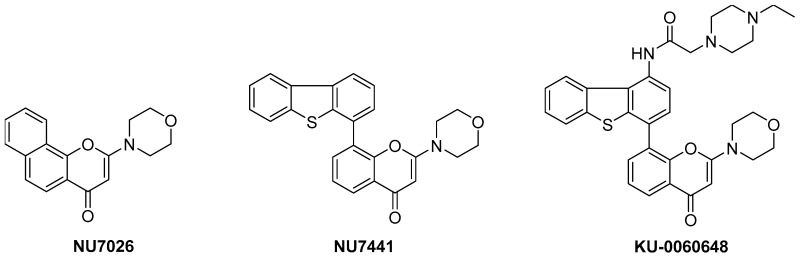


### 2.5. New Dual Antiangiogenic and Anti-Breast Cancer 1-Aryl-3-[3-(thieno[3,2-b]pyridin-7-ylthio)phenyl]ureas (IL5)

QueirozMaria-João R. P.^1^*MachadoVera A.^1^,^2^PeixotoDaniela^1^SoaresRaquel^2^1Department of Chemistry, University of Minho, 4710-057 Braga, Portugal2Department of Biochemistry, Faculty of Medicine, University of Porto, 4200-319 Porto, Portugal*Correspondence: mjrpq@quimica.uminho.pt

Recently, using rational design, we have prepared and discovered a new substitution pattern for potent type II Vascular Endothelium Growth Factor Receptor 2 (VEGFR2) tyrosine kinase inhibitors. These thieno[3,2-*b*]pyridinthiophenyl 1,3-diarylureas **1a–e** are antiangiogenic in enzyme-inhibition (IC_50_ 10–28 nM) and several HUVEC (Human Umbilical Vein Endothelial Cells) assays. The activity was rationalised as type II VEGFR 2 inhibitors based on the simultaneous presence of the phenylthioether linker and the aryl urea moiety in the meta-position. Significant inhibition of proliferation, migration and tube formation of HUVECs was observed at low concentrations and the compounds increased apoptosis in the TUNEL assay. Immunostaining for total and phosphorylated (active) VEGFR2 in HUVECs was performed by Western blotting. The phosphorylation of the receptor was significantly inhibited at 1.0 μM (**1a–c**), 2.5 μM and 5 μM (**1d,e**) by the most promising antiangiogenic compounds. The latter compounds have significant effects at the same concentrations as the standard sorafenib but with better profiles (Machado, V.A., et al. *Bioorg. Med. Chem*. **2015**, *23*, 6497–6509).


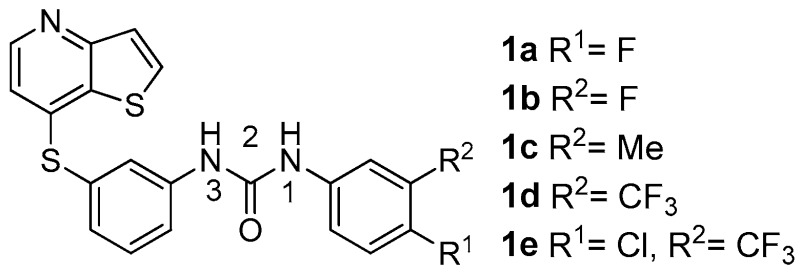


These compounds were also studied against two human breast cancer cell lines of distinct histological types: hormone-dependent MCF-7 (ER positive) and hormone-independent MDA-MB-231 (ER/PR/HER2 negative), the latter resulting from the most aggressive and difficult to treat breast cancer. Our findings demonstrated that **1a**–**e** suppress survival, proliferation, migration and colony formation of breast cancer cells at very low concentrations, while not showing cytotoxicity in normal mammary cells (MCF-10A). TUNEL assays showed that all the compounds induced apoptosis in MDA-MB-231 but not in MCF7 cells at the concentrations tested. PI3K/Akt and MAPK/Erk cell signalling pathways were investigated using Western blot analysis, revealing that the compounds decrease the signalling activity in both breast cancer cell lines. Compounds **1b**, **1c** and **1e** were the most effective particularly in MDA-MB-231 cells. Overall, compounds **1c** and **1e** showed to be the most promising antitumour compounds (Machado, V.A., et al. *J. Cell. Biochem*. **2016**, *117*, 2791–2799).

These findings, together with the antiangiogenic activity, render these compounds a relevant break-through especially for breast cancer therapy (Machado, V.A., et al. *J. Cell. Biochem*. **2016**, *117*, 2791–2799).

**Acknowledgments:** Acknowledgments are due to FCT-Portugal and i3S-University of Porto Portugal.

### 2.6. Fight Against Neglected Tropical Diseases—Arylimidazo[1,2-a]pyrazines as Promising Antileishmanial Agents (IL6)

MarchandPascal^1^*BazinMarc-Antoine^1^PagniezFabrice^2^CojeanSandrine^3^CavéChristian^3^BernadatGuillaume^3^NourrissonMarie-Renée^1^PicotCarine^2^BachStéphane^4^RuchaudSandrine^4^BaratteBlandine^4^LoiseauPhilippe^3^Le PapePatrice^2^1Department of Medicinal Chemistry, IICiMed—EA1155, IRS2, University of Nantes, 44200 Nantes, France2Department of Parasitology and Medical Mycology, IICiMed—EA1155, IRS2, University of Nantes, 44200 Nantes, France3BioCIS—UMR CNRS 8076, Faculty Pharmacy, University Paris-Sud, 92296 Châtenay-Malabry, France4CNRS USR3151, Sorbonne Universities, UPMC Paris 06, Station Biologique, 29680 Roscoff, France*Correspondence: pascal.marchand@univ-nantes.fr

According to a recent report from the World Health Organization (WHO), leishmaniases—visceral leishmaniasis (VL), cutaneous leishmaniasis (CL), and mucocutaneous leishmaniasis (MCL)—collectively affect 12 million people in 98 countries, 350 million more are at risk of infection and 40,000 deaths are attributed to leishmaniases each year (Sangshetti, J.N., et al. *RSC Adv*. **2015**, *5*, 32376–32415). Currently, there are no effective vaccines and a number of drugs are used in the treatment of these parasitic infections: pentavalent antimonials, amphotericin B, miltefosine, pentamidine, paromomycin, and sitamaquine (Sangshetti, J.N., et al. *RSC Adv*. **2015**, *5*, 32376–32415). Unfortunately, most of them cause side effects and high toxicities, and an inevitable resistance has developed in recent times in Leishmania parasites. Consequently, there is an urgent need to speed up the development of a new generation of more effective and safe antileishmanials.


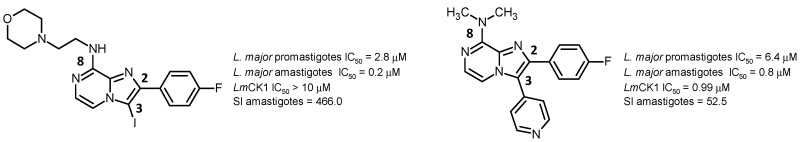


In the course of our ongoing synthetic and screening programs for the obtention of new biologically active imidazo[1,2-*a*]azines, we decided to develop bioisostere analogues of the previously described 2,3-diarylimidazo[1,2-*a*]pyridines as antileishmanial agents (Marhadour, S., et al. *Eur. J. Med. Chem.* **2012**, *58*, 543–556).

Two promising analogues were highlighted as hit compounds, exhibiting very good activity and high therapeutic index, especially on amastigote stage of the parasite (Marchand, P., et al. *Eur. J. Med. Chem*. **2015**, *103*, 381–395).

From these results, further pharmacomodulation will be presented in order to validate the structural requirements for biological activity. The mechanism of action involved in the antiparasitic properties will be discussed since 4-pyridyl moiety at the C-3 position of the heterocyclic core was associated with *L. major* Casein Kinase 1 (*Lm*CK1) inhibition, validated as potential molecular target for antileishmanial drug development (Durieu, E., et al. *Antimicrob. Agents Chemother*. **2016**, *60*, 2822–2833).

### 2.7. From Artemisinin to Tetraoxane-Based Antimalarial Drug Candidates (IL7)

O’NeillPaul M.^1^*AmewuRichard K.^1^SabbaniSunil^1^ShoreEmma L.^1^RobertsNatalie L.^1^WongMichael H. L.^1^NixonGemma^1^BiaginiGiancarlo A.^2^IsmailHanafy^2^WardStephen A.^2^1Department of Chemistry, University of Liverpool, Liverpool L69 7ZD, UK2Liverpool School of Tropical Medicine, Pembroke Place, Liverpool L3 5QA, UK*Correspondence: pmoneill@liv.ac.uk

Currently, the semi-synthetic artemisinin derivatives artesunate and artemether are the mainstay of malaria chemotherapy but their use is hampered by supply and cost. Most people needing treatment for malaria cannot afford drugs containing artemisinin; nevertheless, artemisinin and its derivatives remain the most effective antimalarials and are currently used in combination with other drugs as recommended by the World Health Organization (WHO). The first part of the presentation will focus on mechanistic studies designed to unravel the antimalarial mechanism of action of the endoperoxide class of drug (Ismail, H.M., et al. *Proc. Natl. Acad. Sci. USA* **2016**, *113*, 2080–2085; O’Neill, P.M., et al. *Angew. Chem. Int. Ed*. **2010**, *49*, 5693–5697; Stocks, P.A., et al. *Angew. Chem. Int. Ed.* **2007**, *46*, 6278–6283). These studies include biomimetic studies with Fe(II) haem and chemical proteomic approaches.


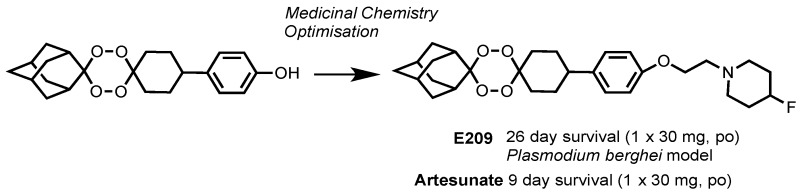


Recently, resistance has developed to the artemisinins in *Plasmodium falciparum* parasites through mutations in the pfKelch13 gene. It is proposed that the resistance mechanism enables the malaria parasites to mount an oxidative stress response. The final part of the presentation will describe a collaborative, multinational, drug discovery programme that has delivered a novel fully synthetic tetraoxane-based molecule, E209, that meets the requirements of the Medicines for Malaria (MMV) target drug candidate profiles (O’Neill, P.M., et al. *Nat. Commun*. **2017**, *8*, 15159). Importantly, E209 displays minimal cross resistance against parasites carrying mutations in pfKelch13 that mediate resistance to dihydroartemisinin. E209 has potent nanomolar inhibitory activity against multiple strains of *P. falciparum* and *P. vivax* in vitro and is efficacious against *P. falciparum* in in rodent models in vivo, produces parasite reduction ratios equivalent to dihydroartemisinin and has pharmacokinetic and pharmacodynamic characteristics that are compatible with a single-dose cure. 

### 2.8. Towards an Alternative to NSAIDs Based on Vitamin E Analogues (IL8)

HélesbeuxJean-Jacques^1^*AlsabilKhaled^1^VilleAlexia^1^Suor-ChererSorphon^1^ViaultGuillaume^1^TemmlVeronica^3^KoeberleAndreas^2^SchusterDaniela^3^WerzOliver^2^StuppnerHermann^3^SeraphinDenis^1^RichommePascal^1^1SONAS, SFR4207 QUASAV, University of Angers, 49000 Angers, France2Institute of Pharmacy, Friedrich-Schiller University, 0740 Jena, Germany3Institute of Pharmacy/Pharmacognosy, University of Innsbruck, 8020 Innsbruck, Austria*Correspondence: jj.helesbeux@univ-angers.fr

Inflammation is the immune system’s response to infection and injury. It is an intrinsically beneficial event that leads to the removal of offending factors and the restoration of tissue structure and physiological function. Once the initiating noxious stimulus is removed, the inflammatory reaction can decrease and resolve. However, in the case that acute inflammatory processes persist, a chronic inflammatory state occurs. Chronic inflammation is implicated in the pathogenesis of many diseases including asthma, arthritis, skin diseases, cancer and stroke, as well as in neurodegenerative and cardiovascular diseases (Medzhitov, R. *Nature* **2008**, *454*, 428–435). This process is driven by pro-inflammatory lipid mediators, i.e., prostaglandin (PG)E2 and leukotrienes (LTs), biosynthesised from arachidonic acid (AA). As the 5-lipoxygenase (5-LOX) is involved in the first two stages of the metabolism from AA to LTs, this enzyme has been described as a key target in the development of new anti-inflammatory drugs (Radmark, O., et al. *Trends Biochem. Sci*. **2007**, *32*, 332–341). As a matter of fact, the current long term anti-inflammatory treatment based on the use of non selective or selective non-steroidal anti-inflammatory drugs (NSAIDs) is often associated with severe side effects. Therefore there is a crucial need for new molecules to complete the therapeutic arsenal against inflammation, potentially through the efficient inhibition of the production of LTs.

An in silico screening associated to a focused biological evaluation led us to the identification of new tocotrienolic leads exhibiting an IC_50_ against 5-LOX below 200 nM (Richomme, P., et al. WO2017032881A1, 2 March 2017). Thus to optimize the activity, through the increase of the structural diversity of these class of secondary metabolites and also to explore their mechanism of action, we developed various semisynthetic strategies leading to new analogues whose inhibitory potential of 5-LOX activity has been evaluated.

### 2.9. Targeted Drug Discovery Applied to Unmet Medical Need in Metastatic Breast Cancer (IL9)

BordoniCinzia^1^SoukupovaJitka^1^PiggottLuke^1^GrucaAleksandra^2^TurnhamDaniel^2^YangWill^2^ClarksonRichard W. E.^2^BrancaleAndrea^1^WestwellAndrew D.^1^*1School of Pharmacy and Pharmaceutical Sciences, Cardiff University, Cardiff CF10 3NB, UK2European Cancer Stem Cell Research Institute, School of Biosciences, Cardiff University, Cardiff CF24 4HQ, UK*Correspondence: WestwellA@cf.ac.uk

Advances in breast cancer research have established the existence of clinical disease sub-types, each with distinct pathologies, course of disease progression, and response to therapeutic intervention. Survival outcomes have improved dramatically in recent years for oestrogen receptor (ER)-positive disease, largely due to the routine use of tamoxifen or related anti-oestrogens such as anastrozole. On the other hand, significant disease sub-types such as “triple-negative” breast cancer (lacking expression of ER, progesterone or HER2 receptor; around 15% of cases), present a difficult challenge with standard chemotherapy having little impact on overall survival. In addition, treatment of HER2-positive breast cancer, a sub-type characterised by frequent metastatic progression, is also clinically challenging. Treatment of HER2-positive disease can be partially addressed by agents such as the monoclonal antibody trastuzumab, albeit with disease relapse and progression in many cases.

Previous research at Cardiff has established the important role of Bcl3 in metastatic progression of HER2-positive breast cancer within models in vitro and in vivo (Wakefield, A., et al. *Cancer Res*. **2013**, *73*, 745–755). Bcl3 is a facilitator protein of the NF-κB signalling system with significant potential as a target for cancer drug design.

At the outset of this project, there were no reported inhibitors of Bcl3, however previous structural biology studies had established crystal structures for Bcl3 and protein binding partners. We chose to focus on the Bcl3-p50 protein-protein interaction, generating a pharmacophore model for virtual screening. Subsequent docking and refinement of virtual hit compounds led to the identification of ten distinct compounds for evaluation in vitro. One of these compounds (JS6) exhibited potent (sub-micromolar) inhibitory activity in a range of relevant breast cancer models including an NF-κB reporter cell line and in cell migration assays. Crucially, JS6 effectively suppressed metastatic progression in in vivo models bearing human metastatic MDA-MB-231 cells. These results have led to patent filing, and subsequent licensing to a commercial partner. Further pre-clinical development studies are ongoing and will be discussed.

### 2.10. Discovering Bioactive Products from Lichen Symbiotic Network (IL10)

TomasiSophieUMR CNRS 6226 ISCR Equipe CORINT Univ. Rennes 1, Université Bretagne Loire, 2 Avenue du Pr. Léon Bernard, F-35043 Rennes, France; sophie.tomasi@univ-rennes1.fr

Long-living symbioses are particularly rich bioresources of novel bioactive metabolites. Lichens are self-supporting symbiotic associations formed by a fungus and one or several algal components as primary partners but also provide an ecological niche for a high diversity of additional bacteria (Cardinale, M., et al. *FEMS Microbiol. Ecol*. **2008**, *66*, 63–71; Bjelland, T., et al. *Environ. Microbiol. Rep.* **2011**, *3*, 434–442; Bates, S.T., et al. *Appl. Environ. Microbiol*. **2011**, *77*, 1309–1314; Grube, M., et al. *Microbiol. Spec.* **2016**, *4*, 1–17). Lichens are well known for their ability to produce a diversity of different bioactive compounds (Boustie, J., et al. *Phytochem. Rev*. **2011**, *10*, 287–307). They have evolved effective means to control their inhabitants by producing metabolites acting as a communication and controlling system. Shrestha and St Clair have thus reported the antibiotic activity of a number of lichen metabolites (Shrestha, G., et al. *Phytochem. Rev*. **2013**, *12*, 229–244). Moreover, in mutualistic symbiosis, microorganisms evolved to produce small bioactive metabolites that guide the composition of the whole association (holobiont). In this context lichen-associated bacteria have been already reported as a promising source of highly effective compounds such as uncyalamycin, cladoniamides, aminocoumarines … (Suzuki, M.T., et al. *Appl. Microbiol. Biotechnol.* **2016**, *100*, 583–595; Parrot, D., et al. *Sci. Rep*. **2015**, *2348*, 1–14).

An overview of our work will be exposed, focusing on the discovery of bioactive metabolites isolated from lichens and/or their associated bacteria and acting as cytotoxic or antibiotic compounds.


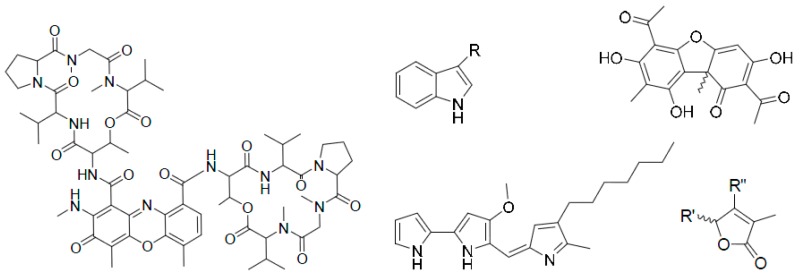


**Acknowledgments:** We thank the financial support of CNRS, ANR Malica, Ligue Contre le Cancer 35, to the CORINT team.

### 2.11. 1,3-Aminoalcohols: Introduction of Three Chiral Centres in One Pot (IL11)

FoleyVeraMcGlackenGerard*Department of Chemistry and the Analytical and Biological Chemistry Research Facility (ABCRF), University College Cork, Cork, Ireland*Correspondence: g.mcglacken@ucc.ie

The 1,3-aminoalcohol moiety is present in many molecules of biological interest and in chiral ligands (Lait, S.M., et al. *Chem. Rev*. **2007**, *107*, 767–796). Methods for the preparation of 1,3-aminoalcohols and their derivatives usually involve a two-step installation of the chiral centres (e.g., Kochi, T., et al. *J. Am. Chem. Soc.* **2002**, *124*, 6518–6519). We envisaged that an aldol-Tishchenko reaction using an imine derivative could provide these valuable synthons asymmetrically.


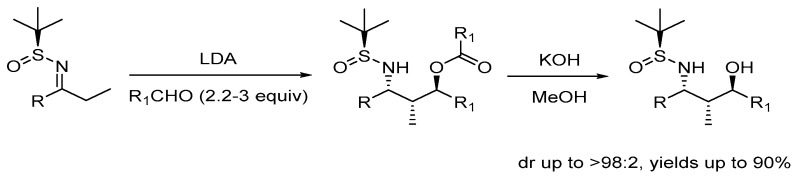


Thus, an aldol-Tishchenko reaction of chiral sulfinimines, which involves the first reported reduction of a C=N in this type of reaction, is described. Two and even three chiral centres can be installed simultaneously in one synthetic step, affording anti-1,3-aminoalcohols in good diastereoselectivity and enantioselectivity (Foley, V.M., et al. *Org. Lett*. **2015,** *17*, 5642–5645).

### 2.12. From Selective, Bivalent Inhibitors to Multi-Target Drugs by the Example of Human Protein Kinase CK2, an Emerging Cancer Target (IL12)

JoseJoachim^1^*Le BorgneMarc^2^NiefindKarsten^3^1Institute of Pharmaceutical and Medicinal Chemistry, Pharma Campus, Westfälische Wilhelms-Universität Münster, 48149 Münster, Germany2Faculté de Pharmacie—ISPB, Université Claude Bernard Lyon 1, EA 4446 Bioactive Molecules and Medicinal Chemistry, SFR Santé Lyon-Est, F-69373 Lyon CEDEX 8, France3Department of Chemistry, Institute of Biochemistry, University of Cologne, 50674 Köln, Germany*Correspondence: joachim.jose@uni-muenster.de

Protein kinase CK2 is a member of the eukaryotic protein kinases (EPK) and consists of two catalytic α-chains attached to a dimer of regulatory β-subunits. CK2 is a regulator of cellular key processes and tumor cells overexpress and exploit the enzyme to evade apoptosis. The ATP-site allows the design of high-affinity ligands, e.g., silmitasertib, a quinoline derivative currently in clinical Phase 2 trial for a combinatorial treatment of cholangiocarcinoma. However, due to its structural conservation, the ATP-site offers limited options to generate selectivity. Vice versa non-ATP sites—in CK2, the CK2α/β-interface, the substrate-binding site as well as a recently detected αD-pocket—typically select their ligands pre-cisely but bind them with lower affinity. To combine affinity with selectivity, bivalent inhibitors were developed, addressing the ATP-site and a second cavity, e.g., ARC-1502, a hybrid molecule of 4,5,6,7-tetrabromo benzimidazole and an acidic peptide connected via an octanoic acid linker (Enkvist, E., et al. *Org. Biomol. Chem*. **2012**, *10*, 8645–8653).

For a few years, insight has been growing that addressing multiple targets could be beneficial for thera-peutic intervention. As such it is reflecting the multifactorial genesis of disease and re-echoing the observation of a sometimes poor correlation between in vitro drug effects and in vivo efficacy. It resulted in the concept of polypharmacology and the rational of multi-target drugs. In consequence, kinase inhibitors are no longer termed “dirty drugs” but rather drugs of rich pharmacology. In theory, knowing all of the approx. 3000 drug targets available in humans, in combination with suitable test assays, would enable to determine the overall target–related effects of a compound. This is still dreams of the future. Nevertheless, employing the tools currently at hand, we could show the optimization of lead structures for multi-target effects, by the example of CK2 and its lead indeno[1,2-*b*]indole.

By different substitutions at the A, C, and D ring, it was possible to shift potent CK2 inhibitors into selective ABCG2 inhibitors and vice versa (Gozzi, C., et al. *J. Med. Chem*. **2015**, *58*, 265–277). The best dual compound belonged to the indeno[1,2-*b*]indole-9,10-dione group and contained an N^5^ Isopropyl substituent at the C ring and a methylbutenyloxy group at position-3 of the A ring. It showed an IC_50_ value of 25 nM for CK2 and of 3 μM to the breast cancer-related multiple drug transporter ABCG2. It completely inhibited proliferation of breast cancer cells after incubation for 24 h.

Dual inhibitors of CK2 and the cancer related phosphatase CDC25 were obtained by the use of the indeno[1,2-*b*]indoloquinone scaffold (Alchab, F., et al. *J. Enzym. Inhib. Med. Chem*. **2016**, *31*, 25–32). The best dual inhibitor, with IC_50_ 2.0 μM for CDC25A/B and 4.76 μM for CK2, contained an isopropyl substituent at N^5^ of ring C and a Me group at position-8 of ring D. It was not cytotoxic against three non-neoplastic cell lines and showed moderate activity against ABCG2. 

**Acknowledgments:** The authors gratefully acknowledge numerous valuable contributions of their lab.-members to this work.

## 3. Proffered Talks by Early-Career Researchers

### 3.1. A Natural Products Story: Two Examples in the Alkaloids and Siderophore Series (PT1)

GérardStéphane^1^*BarberotChantal^1^MoniotAurélie^2^Allart-SimonIngrid^1^TrochainArnaud^1^MalleretLaurette^3^BentaherAzzaq^3^HénonEric^1^VélardFrédéric^1^SapiJanos^1^1Institut de Chimie Moléculaire de Reims (ICMR), Université de Reims Champagne-Ardenne, UMR CNRS 7312, UFR Sciences, Moulin de la housse and UFR Pharmacie, 51 rue cognacq-jay, 51096 Reims, France2EA 4691 Biomatériaux & Inflammation en site OSseux (BIOS), Université de Reims-Champagne-Ardenne, UFR Pharmacie, 51 rue cognacq-jay, 51096 Reims, France3Centre International de Recherche en Infectiologie (CIRI), Faculté de Médecine Lyon-Sud, 165 chemin du Grand Revoyet, 69921 Oullins, France*Correspondence: stephane.gerard@univ-reims.fr

In the recent years, cyclic nucleotide phosphodiesterase type 4 (PDE4), which controls intracellular level of cyclic nucleotide cAMP, has emerged as a suitable target for anti-inflammatory therapy (e.g., Mulhall, A.M., et al. *Expert Opin. Investig. Drugs* **2015**, *24*, 1597–1611; Michalski, J.M., et al. *Clin. Pharmacol. Ther.* **2012**, *91*, 134–142). In continuation of our efforts to develop PDE4 inhibitors for the treatment of respiratory diseases (Sukhorukov, A., et al. *J. Org. Chem*. **2012**, *77*, 5465–5469; Gérard, S., et al. WO 2016066973 A1, 6 May 2016), we will describe the synthesis of a new family of pyridazinone derivatives, corresponding molecular modeling study (Figure 1) and their evaluation as anti-inflammatory agents. Among these derivatives, 4,5-dihydropyridazinone analogs possessed promising activity, selectivity towards PDE4 isoenzyme and were able to reduce IL-8 production.

### 3.2. Supramolecular Receptors for the Recognition and Discrimination of Post-Translationally Methylated Lysines (PT2)

GruberTobiasSchool of Pharmacy, University of Lincoln, Lincoln LN6 7DL, UK; tgruber@lincoln.ac.uk

The understanding of gene regulation plays an important role in epigenetics, which describe the molecular mechanisms by which environmental factors control the switching on and off of gene activities (Biel, M., et al. *Angew. Chem. Int. Ed*. **2005**, *44*, 3186–3216). One of the major mechanisms of epigenetic control is methylation and demethylation of lysine residues in histone proteins. The desire to understand the function of these modifications, promoted the development of artificial molecules that recognize and bind methyllysine (Minaker, S.A., et al. *J. Am. Chem. Soc.* **2012**, *134*, 11674–11680). They are promising for a number of applications, e.g., as reagents in biochemical assays, in cell biology and to inhibit protein-protein interactions (Beshara, C.S., et al. *ChemBioChem* **2010**, *11*, 63–66). Thereby, the discrimination of differently methylated lysines (monomethyl, dimethyl, trimethyl), both as a modified amino acid and in proteins, is of special interest.


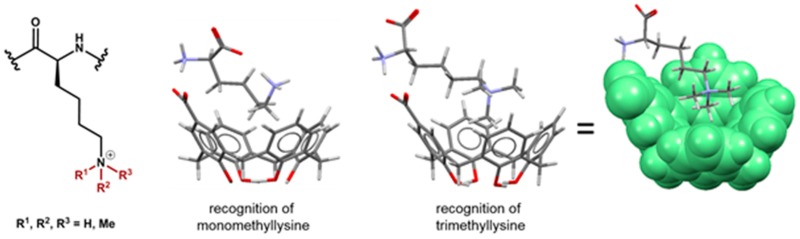


The focus of this presentation is on water-soluble macrocycles and their ability to recognize differently methylated lysines. We will show how respective host molecules discriminate lower methylated lysines, while higher methylated ones bind rather selectively (Hanauer, T., et al. *Org. Biomol. Chem.* **2017**, *15*, 1100–1105). Binding studies were carried out with respectively methylated lysines using NMR spectroscopy and our findings have also been analyzed by energy-minimization calculations. For comparison reason, we included other methylammonium ions like acetylcholine, choline and carnitine in the investigation. By introducing a condensed aromatic system, the resulting receptor is, on the one hand, envisaged to enable better C-H···π interactions with methyl groups of a guest and, on the other hand, to quantify host-guest interactions (Bürger, M., et al. *J. Org. Chem*. **2015**, *80*, 4882–4892). We suggested the name ‘fluorescophane’ for such macrocyclic compounds. Being more sensitive than other methods, this kind of artificial receptors lay the foundations for improved biochemical essays and diagnostics for cancer.

### 3.3. Improved Analgesics: BU08028 a Novel, Bifunctional NOP/MOP Ligand (PT4)

Cami-KobeciGerta^1^*KoMei-Chuan^2^TollLawrence^3^HusbandsStephen M.^1^1Department of Pharmacy & Pharmacology, University of Bath, Claverton Down, Bath BA2 7AY, UK2Department of Physiology & Pharmacology, Wake Forest University, Winston-Salem, NC 27109, USA3Torrey Pines Institute for Molecular Studies, Port St. Lucie, FL 34987, USA*Correspondence: g.cami-kobeci@bath.ac.uk

Although mu opioid (MOP) analgesics, such as morphine, are the preferred analgesics, they possess well-characterised, unwanted effects such as respiratory depression and abuse potential.

When a NOP (nociceptin receptor) agonist is co-administered with a MOP agonist, the combination produces a synergistic effect, resulting in enhanced analgesia (Ko, M.C., et al. *ACS Symp. Ser.*, **2013**, *1131*). This suggests the possibility of obtaining strong analgesia with only low efficacy partial agonism at both MOP and NOP receptors. Structure-activity relationship studies suggested that the region of space occupied by the *tert*-butyl group in buprenorphine was key to good NOP receptor activity (CamiKobeci, G., et al. *J. Med. Chem.* **2011**, *54*, 6531–6537; Khroyan, T.V., et al. *J. Pharmacol. Exp. Ther*. **2011**, *336*, 952–961; Cami-Kobeci, G., et al. *Pharmacol. Rep*. **2011**, *63*, 210). We have discovered BU08028, and other compounds, having binding affinity at MOP receptors similar to that of buprenorphine and, as desired, higher affinity and considerably higher efficacy than buprenorphine at NOP receptors.


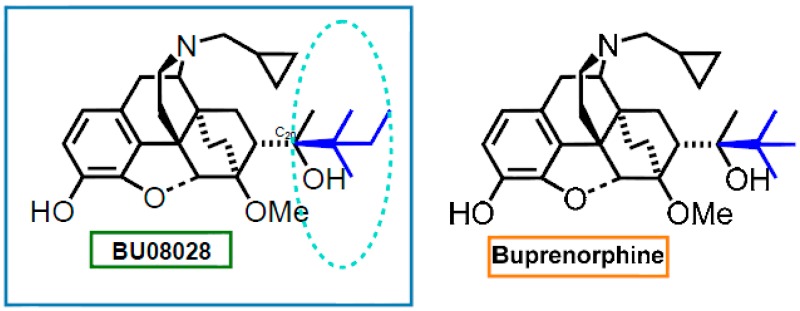


BU08028 has been evaluated in vivo for attenuation of acute pain, using the tail flick assay and these results will be presented.

In measures of hyperalgesia, both in rodents (rats) and nonhuman primates (rhesus monkey) BU08028 was a potent, long-acting anti-hyperalgesic with both MOP and NOP components to its activity. When administered alone in doses up to 0.01 mg·kg^−1^, BU08028 did not induce itch scratching, a standard side-effect of opioids such as buprenorphine (Ding, H., et al. *Proc. Natl. Acad. Sci. USA* **2016**, *113*, 5511–5518). More importantly, BU08028 at antinociceptive doses, did not compromise physiological functions including respiration and cardiovascular activities measured using radio-telemetric probes.

This study strongly supports the therapeutic potential of ligands with mixed MOP/NOP actions as innovative analgesics in humans.

BU08028 therefore represents a potential analgesic agent, with low side-effect profile. 

### 3.4. From the Design to the In Vitro Evaluation of New Iodinated Diazaphenanthridine for the Development of Potential 5-HT_4_ SPECT Radiotracer (PT5)

BabinVictor^1^*TournierBenjamin^2^DavisAudrey^1^MilletPhilippe^2^CaillyThomas^1^BouillonJean-Philippe^3^FabisFrédéric^1^1CERMN, UFR Sci Pharmaceut, EA 4258, Normandie University, UNICAEN, CERMN, 14000 Caen, France2Département de Santé Mentale et de Psychiatrie, Hôpitaux Universitaires de Genève, Service de Psychiatrie Générale, Unité des Biomarqueurs de Vulnérabilité, Chemin du Petit-Bel-Air, 2 CH-1225 Genève, Switherland3COBRA, UMR 6014, FR 3038, Université de Rouen, INSA Rouen, CNRS, Rue Tesnière, 76821 Mont Saint-Aignan CEDEX, France*Correspondence: victor.babin@unicaen.fr

Serotonin (5-hydroxytryptamine 5-HT) is a neurotransmitter acting on the central and peripheral nervous system through a large variety of receptors subdivided in seven families. The serotonin receptor subtype-4 (5-HT4R) is known to be involved in physiological and pathological disorders such as Alzheimer disease (Lezoualc’h, F. *Exp. Neurol*. **2007**, *205*, 325–329). The development of radiotracer is essential for the evaluation of new drugs targeting 5-HT4R and also for investigation of the receptor involvement in a variety neurodegenerative and neuropsychiatric disorders.


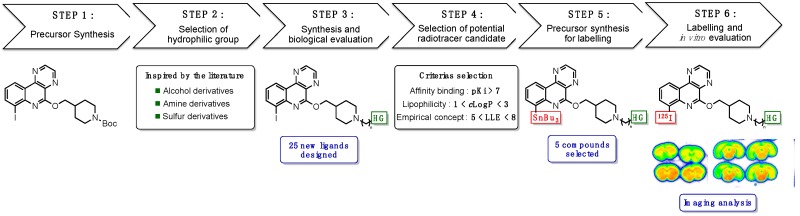


Since a few years, our group has been involved in the development of new SPECT (Single Photon Emission Computed Tomography) radiotracers targeting the 5-HT4R and many iodinated ligands based on phenanthridine (Dubost, E., et al. *J. Med. Chem*. **2012**, *55*, 9693–9707) and diaza-phenanthridine (Fresneau, N., et al. *Eur. J. Med. Chem*. **2015**, *94*, 386–396) scaffolds have been designed. An efficient synthetic access to these compounds has been established and many potent ligands have been synthetized. Biological evaluation has led to a “hit” compound and this ligand and some analogues have been labeled with 125-iodine for SPECT imaging but to date none of these radioligands have been able to image the 5-HT4R specifically. In order to obtain an efficient 5-HT4R radiotracer, our group has decided to synthesize a series of new iodinated ligands with reduced lipophilicity to promote specificity in vivo. The sequence to obtain new radiotracer will be presented in this communication.

### 3.5. Dual-Site-Binding Tankyrase Inhibitors as Potential Agents towards the Treatment of Colorectal Cancer and Type-2 Diabetes (PT6)

NathubhaiAmit^1^*HaikarainenTeemu^2^KoivunenJarkko^2^MurthySudarshan^2^KoumanovFrançoise^3^LloydMatthew D.^1^HolmanGeoffrey D.^3^PihlajaniemiTaina^2^ToshDavid^3^LehtiöLari^2^ThreadgillMichael D.^1^1Department of Pharmacy & Pharmacology, University of Bath, Claverton Down, Bath BA2 7AY, UK2Faculty of Biochemistry and Molecular Medicine, University of Oulu, P.O. Box 5400, 90014 Oulu, Finland3Department of Biology and Biochemistry, University of Bath, Claverton Down, Bath BA2 7AY, UK*Correspondence: a.nathubhai@bath.ac.uk

Based on our previous studies (Nathubhai, A., et al. *ACS Med. Chem. Lett*. **2013**, *4*, 1173–1177; Paine, H.A., et al. *Bioorg. Med. Chem*. **2015**, *23*, 5891–5908; Kumpan, K., et al. *Bioorg. Med. Chem*. **2015**, *23*, 3013–3032; Nathubhai, A., et al. *Eur. J. Med. Chem*. **2016**, *118*, 316–327), we designed and synthesised tankyrase-1 (TNKS1/PARP5a) and tankyrase-2 (TNKS2/PARP5b) inhibitors 1 and 2. Crystal structures of the inhibitors with TNKS2 confirm that the chimeric compounds bind to the nicotinamide and adenosine-binding site as designed (Nathubhai, A., et al. *J. Med. Chem*. **2017**, *60*, 814–820). These dual-site binders were evaluated against eleven PARP isoforms to reveal potent (IC_50_ 1 = 0.1 pM; 2 = 0.2 pM vs. TNKS2) and selective (up to 1 × 10^6^ towards TNKS2) inhibition. Our results place 1 and 2 amongst some of the most potent and isoform-selective TNKS inhibitors known to date. Aberrant Wnt/β-catenin signalling contributes to 90% of colorectal cancers and is regulated by TNKS1/2 (Nathubhai, A., et al. *J. Med. Chem*. **2017**, *60*, 814–820). At the cellular level, both 1 and 2 inhibit Wnt/β-catenin signalling (29–37 nM) and significantly reduce the proliferation of DLD-1 colon adenocarcinoma cells compared to extensively studied commercial TNKS inhibitors (Nathubhai, A., et al. *J. Med. Chem*. **2017**, *60*, 814–820). TNKS1/2 are involved in glucose-uptake but the lack of potent and isoform-selective inhibitors, has hindered pharmacological efforts towards resolving the mechanism of TNKS-mediated glucose uptake (Nathubhai, A., et al. *J. Med. Chem*. **2017**, *60*, 814–820). Our agents demonstrate that potent and isoform-selective TNKS inhibition is essential for insulin-stimulated glucose uptake into adipocytes. Our new TNKS inhibitors may serve as potential drugs to treat cancers that express aberrant levels of Wnt/β-catenin signalling and provide a novel strategy to combat type-2 diabetes. 


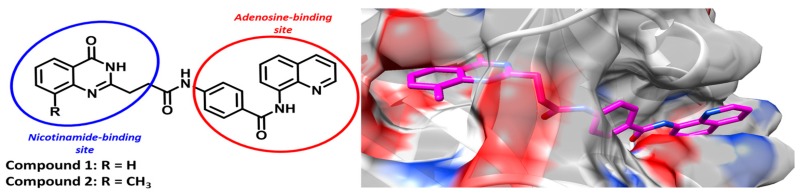


**Acknowledgments:** We thank Worldwide Cancer Research for financial support.

### 3.6. Investigation of Benzofuran-Based Duocarmycins as Chemical Tools to Probe CYP2W1 Functional Activity (PT7)

MoraisGoreti Ribeiro*PresaDanielaNigroGiulianoSheldrakeHelen M.PattersonLaurence H.ShnyderSteveLoadmanPaul M.PorsKlausInstitute of Cancer Therapeutics, Faculty of Life Sciences, University of Bradford, Bradford BD7 1DP, UK*Correspondence: gribeiro@bradford.ac.uk

The cytochromes P450 (CYPs) are a superfamily of haemoproteins responsible for the oxidation of a range of xenobiotics and endogenous compounds. Among the CYP family, the newly identified CYP2W1 is frequently expressed in both primary and metastatic colorectal, adrenocortical, lung, and gastric cancers while absent in healthy tissues. The CYP2W1 expression positively correlates with the degree of malignancy and poor prognosis (Guo, J., et al. *Drug Metab. Rev.* **2016**, *48*, 369–378). CYP2W1 has emerged as a valid drug target and the Pors’s group is engaged in the identification of pharmacophores that are bioactivated by CYP2W1.

The ultrapotency of cytotoxic *seco*-duocarmycins depends on the hydroxy group of the chloromethylindoline fragment to promote spirocyclisation to a cytotoxin that covalently binds N^3^-adenine that leads to DNA damage lethal to the cell. We have shown that re-engineered de-hydroxylated duocarmycin bioprecursors are biologically inactive until bioconverted by CYP2W1 to ultrapotent cytoxins that selectively eradicates colorectal cancer cells in vitro and in vivo (Pors, K., et al. *Chem. Commun*. **2011**, *47*, 12062–12064). We have demonstrated that pyrrolchloromethylindolines are metabolised by CYP2W1 into cytotoxic products (Figure 1, Route A), whereas benzofuran-based duocarmycins are non-cytotoxic (Travica, S., et al. *Clin. Cancer Res*. **2013**, *19*, 2952–2961; Sheldrake, H.M., et al. *J. Med. Chem*. **2013**, *56*, 6273–6277). Here we report on the ongoing investigation of furanindoline-based duocarmycins as CYP2W1 substrates. Metabolic studies with HEK293/mock and HEK293/CYP2W1-transfected cells have shown that the 5′-fluoro analogue, ICT2726, is oxidised selectively by CYP2W1 to generate an innocous metabolite (Route B). While the furanindoline pharmacophore is unsuitable for tumour-selective therapy, the selective CYP2W1 bioactivation of the ICT2726 suggest that this duocarmycin analogue could be used as chemical tool to probe CYP2W1 functional activity.

**Acknowledgments:** This work was supported by a YCR Programme Grant.

## 4. Posters with Flash Oral Presentations

### 4.1. Synthesis and Antiproliferative Action of Novel Ethanoanthracenes: Potential Therapies for Lymphomas and Leukaemias (PF1)

McKeownJames P.ByrneAndrew P.CharletonClara E.*FerrisKeithMeeganMary J.Trinity Biomedical Sciences Institute, 152-160 Pearse Street, Trinity College Dublin, Dublin 2, Ireland*Correspondence: charletc@tcd.ie

The antidepressant maprotiline and its (*E*)-9(2nitrovinyl)anthracene analogues have been identified as potent novel antiproliferative compounds in Burkitt Lymphoma (BL) cell lines MUTU-1 and DG-75 (Cloonan, S.M., et al. *Int. J. Cancer* **2011**, *128*, 1712–1723; McNamara, Y.M., et al. *Eur. J. Med. Chem*. **2014**, *71*, 333–353). This knowledge was translated to CLL (Chronic Lymphocytic Leukaemia), a related B cell malignancy. CLL is the most common leukaemia in the western world, accounting for greater than one third of new diagnoses.

Initial biochemical screening (Alamar Blue) in PGA1 and HG3 CLL cell lines of a series of anthracenes has been completed and a number of potent compounds were identified. The results were then compared to our BL studies, with compounds being tested at 1 μM and 10 μM concentrations. Activity was determined as percentage of viable cells remaining. The subsequent finding of these studies is presented together with discussion of the rationalisation of the compound SAR in the chronic lymphocytic leukaemia PGA1 and HG3 CLL cell lines. Synthesis of (*E*)9-(2nitrovinyl)anthracenes was accomplished through the use of a piperidine-catalysed Henry-Knoevenagel condensation reaction, in addition to Grignard and Vilsmeier-Haack chemistry to yield the required 10-substituted 9-anthraldehyde intermediates. Diels-Alder chemistry affords the ethanoanthracene-type maprotiline analogues (Figure 1).

The expansion of the current SAR knowledge for the ethanoanthracene-type maprotiline analogues and bioisosteric replacement of the nitro group on the 9-vinyl anthracene substituent with α,β-unsaturated ketones with a view to optimising biological activity will be presented, together with the determination of the antiproliferative and pro-apoptotic activities in CLL cell lines.

### 4.2. Synthesis of Original Serotonergic Multi-Target-Directed Ligands—The Triad Program (PF4)

HatatBérénice^1^,^2^*LecouteyCédric^1^YahiaouiSamir^1^DavisAudrey^1^FreretThomas^3^BoulouardMichel^3^ClaeysenSylvie^2^RochaisChristophe^1^DallemagnePatrick^1^1Centre d’Etudes et de Recherche sur le Médicament de Normandie (CERMN)–UPRES EA 4258–FR CNRS INC3M–SFICORE, Université de Caen Normandie, UFR des Sciences Pharmaceutiques–Bd Becquerel, F-14032 Caen, France2CNRS, UMR-5203, Institut de Génomique Fonctionnelle, F-34000 Montpellier, France3Unité COMETE UMR-S 1075 INSERM, 2 rue des Rochambelles, F-14032 Caen, France*Correspondence: berenice.hatat@unicaen.fr

Targeting more than one molecular cause implied in the pathogenesis of Alzheimer’s disease (AD) with a sole drug is considered a promising challenge, because it could address the failures that recently occurred during clinical trials. Within this framework, we recently reported the design of donecopride, a pleiotropic agent that both displays acetylcholinesterase (AChE) inhibition and 5-HT_4_R agonist activity (Lecoutey, C., et al. *Proc. Natl. Acad. Sci. USA* **2014**, *111*, E3825–E3830). Based on its procognitive and antiamnesiant in vivo properties, donecopride is currently under preclinical investigation. A pharmacomodulation study allowed to establish the structure-activity relationships in the donecopride series and to enlarge the latter with some other potent ligands (Rochais, C., et al. *J. Med. Chem.* **2015**, *58*, 3172–3187). Taking into account the clinical interest of idalopirdine, a 5-HT_6_R antagonist, in Alzheimer’s Disease (AD) treatment, we undertook a new program aiming at designing novel Multi-Target Directed Ligands targeting both but selectively AChE, 5-HT_4_ and 5-HT_6_ receptors.

Considering the pharmacophores established for each of the three targets, we performed the synthesis of numerous donecopride derivatives. Among them, some dual compounds both exhibited 5-HT_4_R agonist and 5-HT_6_R antagonist activities in vitro and displayed a procognitive effect in vivo.

Pursuing the pharmacomodulation of these compounds led to first pluripotent derivatives with in vitro submicromolar activities towards the three designated targets.

The TRIAD program, funded by the Ligue Européenne contre la Maladie d’Alzheimer and the French Fondation Plan Alzheimer, recently led to some novel promising agents with in vitro prerequisite for further evaluation in vivo in AD experimental models.

### 4.3. Human Hyal 1—From In Silico Pharmacophore Modeling to In Vitro Inhibitor Screening (PF5)

LengersIsabelle^1^*HermannFabian^2^HaiderSamer^1^JoseJoachim^1^1Institute of Pharmaceutical and Medicinal Chemistry, PharmaCampus, Westfälische Wilhelms-Universität, Corrensstraße 48, 48149 Münster, Germany2Institute of Pharmaceutical Biology and Phytochemie, PharmaCampus, Westfälische Wilhelms-Universität, Corrensstraße 48, 48149 Münster, Germany*Correspondence: isabelle.lengers@uni-muenster.de

The endoglycosidase hydrolase Hyaluronidase 1 (Hyal 1) is one of five functional hyaluronidases in human body. Degradation of high molecular weight hyaluronan (HA) is mainly catalyzed by Hyal-1 into smaller fragments (Figure 1). These fragments have inflammatory and angiogenic effects (Stern, R. *Semin. Cancer Biol*. **2008**, *18*, 275–280). The role of Hyal-1 in cancer progression, e.g., prostate or bladder, has been discussed for a long time. In several cancer cells, the expression level of Hyal-1 was elevated in comparison to not malignant cells, resulting in higher Hyal 1 activity and tumor progression (Lokeshwar, V.B., et al. *J. Urol.* **2000**, *163*, 348–356; Lokeshwar, V.B., et al. *J. Biol. Chem.* **2001**, *276*, 11922–11932). Although Hyal 1 is an interesting target for pharmaceutical purposes, no potent inhibitors have been found so far. The enzyme source seems to be the bottleneck in investigation of potent inhibitors. Production of active Hyal 1 is one of the most challenging tasks. Eukaryotic extraction and purification is very time consuming and expensive. Recombinant expression in bacteria leads to inactive Hyal-1 forming inclusion bodies. Therefore, potent Hyal-1 inhibitors, like chemical compounds or plant extracts, are routinely screened against bovine testis hyaluronidase, which has an amino acid sequence identity of approx. 80% compared to human Hyal 1. This again causes problems in interpretation of the obtained data, development of a pharmacophore model or searching for leader compounds inhibiting human Hyal 1. Using Autodisplay technology, we are able to express human Hyal-1 on the surface of Escherichia coli in an active form (Orlando, Z., et al. *Molecules* **2015**, *20*, 15449–15498). With this system, it is possible to screen compounds, directly using the desired target. A combination of pharmacophore modeling, followed by docking studies using a virtual system, helped us to get first impressions about binding of the substances to Hyal-1. Next, screening the best hits with whole-cells displaying Hyal-1 seems to be a promising way to find the needle in the haystack.

### 4.4. Differences Detected in Glycosylation Critical Quality Attributes of Epoetin Alpha Biosimilars (PF7)

ThomsonRebecca I.^1^*GardnerRichard A.^2^StrohfeldKatja^1^FernandesDaryl L.^2^StaffordGraham P.^3^SpencerDaniel I. R.^2^OsbornHelen M. I.^1^1Reading School of Pharmacy, University of Reading, Reading RG6 6AP, UK2Ludger Ltd., Culham Science Centre, Abingdon OX14 3EB, UK3Integrated BioSciences, School of Clinical Dentistry, University of Sheffield, Sheffield S10 2TA, UK*Correspondence: r.i.thomson@pgr.reading.ac.uk

When designing a glycoprotein-based biotherapeutic, glycosylation critical quality attributes (GCQAs) must be considered, as they are known to influence the quality, safety and efficacy. This is particularly relevant in the case of biosimilars because the extent and type of post-translational glycosylation can differ greatly according to variation in manufacturing processes (Tsiftsoglou, A.S., et al. *BioDrugs* **2013**, *27*, 203–211). Thus, three erythropoiesis stimulating agents (ESAs), namely Eprex, Binocrit and a development ESA here called CIGB-EPO, were subjected to 1,2-diamino-4,5-methylenedioxybenzene (DMB) sialic acid (SA) labelling, procainamide (PROC) *N*-glycan labelling (Figure 1) and *O*-acetylesterase (NanS) digestion (Hara, S., et al. *J. Chromatogr*. **1986**, *377*, 111–119; Kozak, R.P., et al. *Anal. Biochem.* **2015**, *486*, 38–40; Phansopa, C., et al. *Biochem. J.* **2015**, *472*, 157–167.). Samples were analysed using liquid chromatography-mass spectrometry (LC-MS) or LC alone. This combination of methods highlighted previously undetected differences in GCQAs among the three biotherapeutics. Notably, Eprex was found to contain the greatest relative abundance of O-acetylated SA derivatives, Binocrit the lowest level of the immunogenic SA derivative *N*-glycolylneuraminic acid (Neu5Gc), and CIGB-EPO showed the greatest variety of high-mannose-phosphate structures. The sialylation and *N*-lactosamine (LacNAc) extension patterns of the three products were similar, with a maximum of four *N*-acetyl-neuraminic acid (Neu5Ac) moieties detected per glycan (Thomson, R.I., et al. *Anal. Chem*. **2017**, *89*, 6455–6462). In order to understand better the structure-activity relationships (SARs) of ESAs and to tune their quality, safety and efficacy, it is suggested that ESAs under development be subjected to the same level of analysis. 

## 5. Posters

### 5.1. Investigating Efficient Synthetic Routes to Novel Narciclasine Derivatives (P3)

AzubuikeDarlington*CaggianoLorenzoDepartment of Pharmacy & Pharmacology, University of Bath, Claverton Down, Bath BA2 7AY, UK*Correspondence: d.o.azubuike@bath.ac.uk

Narciclasine is a natural product obtained from the bulbs of various *Amaryllidaceae* plants, such as daffodils (e.g., Rinner U., et al. *Synlett* **2005**, *3*, 365–387; Kornienko, A., et al. *Chem. Rev.* **2008**, *108*, 1982–2014). It is of great interest due to its potent anti-tumour properties, in particular against primary brain tumours. Complex total synthesis and low-yielding extraction from daffodil bulbs have limited their progression to preclinical and clinical research. Our research group has already established synthetic methodology to generate the narciclasine core in a single high-yielding step (Judd, K.E., et al. *Synthesis* **2009**, *16*, 2809–2817). We will apply this methodology to the synthesis of novel narciclasine analogues and explore their activities. Of interest is that the structurally related derivative narciprimine has also been isolated from daffodil bulbs; however, it displays little or no biological activity. We therefore wish to synthesise efficiently novel narciclasine/narciprimine hybrid analogues and study their activities, which will increase our understanding relating to the activity of narciclasine and inactivity of narciprimine. Most importantly, we will examine if the aromaticity of the C-Ring and stereochemistry is necessary for activity.

We have now established a highly efficient Suzuki cross coupling reaction with both nitrile and ester substrates and obtained the A–C biphenyl group. We are currently pursuing an efficient cyclisation methodology which would yield the narciclasine/narciprimine substructure. In addition, we will synthesise radiolabeled hybrid analogues of narciclasine and narciprimine and perform studies in vivo using Positron Emission Tomography (PET) Imaging.


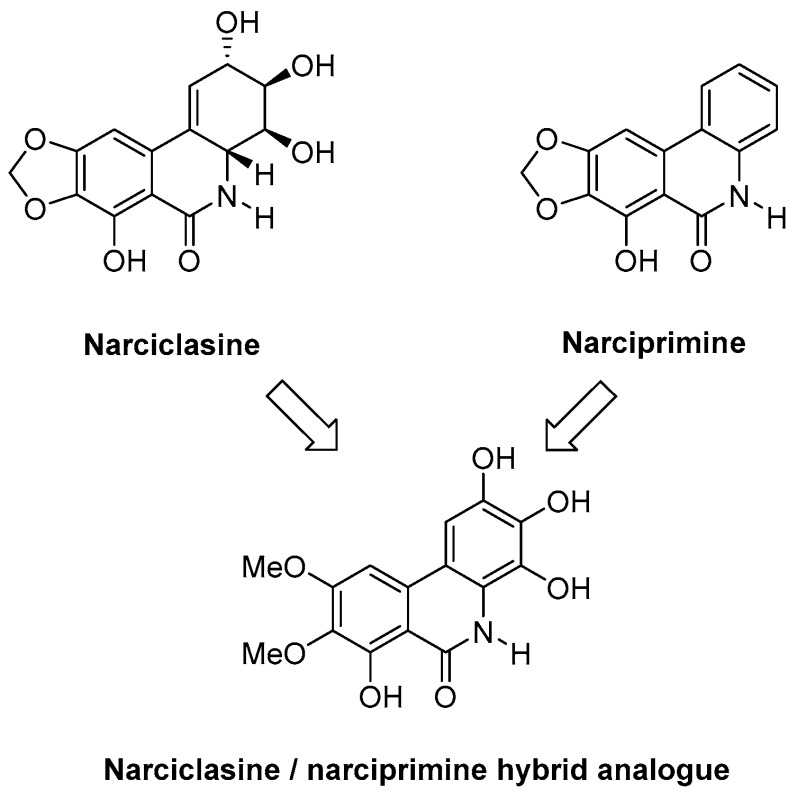


### 5.2. FXR Agonists for Application in Cancer Treatment (P4)

BozIrene^1^*BicknellKatrina^1^Weymouth-WilsonAlex^2^GrecoFrancesca^1^OsbornHelen^1^1Department of Pharmacy, University of Reading, Reading RG6 6AH, UK2NZP UK Ltd., Science and Technology Centre, Reading RG6 6BZ, UK*Correspondence: i.boz@pgr.reading.ac.uk

Bile acids (BAs) are naturally occurring cholesterol derivatives with amphiphilic structure. BAs have been associated with tumour modulation via either inhibiting or inducing activity (Baptissart, M., et al. *Biochimie* **2013**, *95*, 504–517) and different natural BAs have shown specific interactions with a variety of pathways (Figure 1a) (Schaap, F.G., et al. *Nat. Rev. Gastroenterol. Hepatol*. **2014**, *11*, 55–56). For example, BAs interact with the nuclear Farnesoid X Receptor (FXR), known to regulate BA synthesis. FXR expression is documented in high levels in healthy tissues, such as liver, small intestine and kidney; and in lower levels in colon and breast tissues. FXR expression has also been reported in a range of cancerous tissues and cell lines, where it is often found in lower levels, compared to the healthy tissues (Figure 1b) (Vaquero, J., et al. *Biochem. Pharmacol*. **2013**, *86*, 926–939). Thus, FXR could be a promising target for different cancers treatment and besides FXR modulation might be useful in preventing cancer development. 

In this study, the IC_50_ values of the non-steroidal FXR agonist GW4064, and the steroidal FXR agonist obeticholic acid (OCA), have been determined against the hepatocellular carcinoma cell line HuH-7, the colon cancer cell line HCT-116 and the breast cancer cell lines ER+ MCF-7 and triple negative MDA-MB-231. Additionally, a library of BAs has been tested at a single concentration to evaluate the cytotoxicity against the cell lines. These results, together with Quantitative Structure-Activity relationship (QSAR) studies, direct the future design and synthesis of further BAs with FXR modulator activity.

**Acknowledgments:** IB thanks the University of Reading and NZP UK Ltd. for financial support.

### 5.3. Access to New Diagnostic Tools in Cancerology through C-H (Radio-)iodination of N-Acylsulfonamides (P6)

HebertAlexandra*BenoistFlorianDubostEmmanuelleFabisFrédéricCaillyThomasCentre d’Etudes et de Recherche sur le Médicament de Normandie, CERMN UNICAEN EA 4258 FR CNRS 3038 INC3M SF 4206 ICORE Boulevard Becquerel, University of Caen Normandie, Caen 14032, France*Correspondence: alexandra.hebert@unicaen.fr

Labelling of (bio)molecules with radioactive isotopes is of high interest to the scientific community, as it strongly impacts the discovery process in life science and nuclear medicine. Radiolabelled molecules have been extensively used to assess biochemical reactions, to measure in vivo distribution of a substance or to perform RIA (RadioImmunoAssay). In nuclear medicine, radio-therapeutics for RIT (RadioIsotope Therapy) and radio-tracers for molecular imaging experiments such as PET (Positron Emission Tomography), SPECT (Single Photon Emission Computed Tomography) or scintigraphy have been described. Several useful isotopes of iodine can be used for both diagnosis and therapy: ^123^I for SPECT imaging, ^124^I for PET imaging, ^125^I for biological assays and nuclear medicine and ^131^I for radiotherapy and scintigraphy. Our group has recently developed a method to radio-iodinate *N*-acylsulfonamides through a room temperature palladium mediated C-H radio-iodination (Figure 1). This original strategy allows radiolabelling in very mild conditions without the use of chemical precursors.

In this context, we are currently enlarging the scope of this methodology toward the radio-iodination of antitumoral agents containing a *N*-acylsulfonamide group. Thus, an antiproliferative agent LY32262 (Lobb, K.L., et al. *J. Med. Chem*. **2004**, *47*, 5367–5380), a Bcl-xL/Mcl1 dual inhibitor (Rega, M.F., et al. *J. Med. Chem.* **2011**, *54*, 6000–6013) and ABT-737 (Oltersdorf, T., et al. *Nature* **2005**, *435*, 677–681) have been selected and progress toward the (radio-)iodination of these molecules will be presented.

### 5.4. Synthesis and Evaluation of a Range of Seco-Amino-CBIs as Highly Cytotoxic DNA Alkylating Agents (P7)

KennyMichael B. C.*ThompsonAndrew S.LloydMatthew D.ThreadgillMichael D.Department of Pharmacy & Pharmacology, University of Bath, Claverton Down, Bath BA2 7AY, UK*Correspondence: m.kenny@bath.ac.uk

Duocarmycin SA 1 is a natural product of the CPI class with exquisitely potent cytotoxicity (IC_50_ = 10 pM in L1210 cells) (Ichimura, M., et al. *J. Antibiot*. **1990**, *43*, 1037–1038). It binds to the minor groove of DNA, and alkylates using the strained spirocyclopropane ring. Denny and others have shown that 2 is similarly super-potent; in this compound, the exocyclic carbonyl is replaced by an imine and the pyrrole ring is replaced by a second benzene ring, leading to the class being named amino-CBI (Atwell, G.J., et al. *J. Org. Chem*. **1998**, *63*, 9414–9420). The imine functionality can be exploited to allow for the development of a targeted pro-drug whereby a *seco*-amino-CBI is released at the site of a tumour and then undergoes a rapid Winstein cyclisation to form the active amino-CBI.

This work outlines a short, efficient route to a range of seco-amino-CBI analogues (Figure 1). Starting from commercially available 1-hydroxy-napthoic acid, intermediate **3** can be synthesised in 8 steps. At this point in the synthesis, a range of minor-groove binding subunits can be integrated after deprotection of the amine. This key intermediate is then transformed to the final seco-amino-CBIs, **4**–**6**, in a further 3 steps.

### 5.5. Sphingosine Kinase Inhibition—“New Target, Multiple-Cancers” (P8)

KruschelRyan D.^1^*WaeberChristian^2^McCarthyFlorence O.^1^1Department of Chemistry, Analytical and Biological Chemistry Research Facility, University College Cork, Cork, Ireland2Department of Pharmacology and Therapeutics, School of Pharmacy, University College Cork, Cork, Ireland*Correspondence: r.kruschel@umail.ucc.ie

Sphingosine kinase has been described as ‘the golden sword of Hercules’ in terms of its anti-cancer potential (Pyne, S., et al. *Cancer Res*. **2011**, *71*, 6576–6582). Current cancer chemotherapy is inadequate due to dose-limiting side effects. A new target, sphingosine kinase-1 (SK1), has been implicated in the process that controls whether an abnormal cell becomes malignant or is simply allowed to die naturally. SK1 catalyses the phosphorylation of the biolipid sphingosine to an active second messenger, sphingosine-1-phosphate (S1P), whose intracellular levels play a role in determining cell fate (Plano, D., et al. *J. Med. Chem*. **2014**, *57*, 5509–5524; Waeber, C., et al. *Circ. J.* **2014**, *78*, 795–802). An accumulation of S1P leads to cancer cell survival whereas an inhibition leads to apoptosis and cell cycle arrest. Elevated S1P levels are observed in many cancers including breast and prostate. Its concentration in vivo is exploited as a clinical tool in cancer prognosis (Ruckhäberle, E., et al. *Breast Cancer Res. Treat.* **2008**, *112*, 41–52).

Sphingosine (Figure 1) has three definable regions, a polar head, linker and lipophilic tail, all of which are necessary for effective binding in the narrow J-channel located in the active site (Adam, D. R., et al. *Trends Biochem. Sci*. **2016**, *41*, 395–409). Within the last ten years, these regions have been mapped on various chemical scaffolds to generate active inhibitors (Pitman, M. R., et al. *Cell. Signal.* **2016**, *28*, 1349–1363). Isoquinolines show well documented anticancer activity and we build on preliminary evidence to develop lead molecules with specific substitution patterns to influence cancer cell growth. A general isoquinolinequinone scaffold has been developed to provide a versitile starting point to generate novel quinone-based inhibitors.

The isoquinolinequinone scaffold allows functionalisation on a number of different handles to probe binding. The mapping of the three sphingosine regions has lead to the generation of a library of novel isoquinolinequinone derived compounds. Molecular modelling revealed a number of compounds with high binding affinity in silico for SK1 in reference to known SK1-specific inhibitors.

### 5.6. The Synthesis and Biological Testing of Novel Carbohydrate-Based Antibacterial Prodrugs (P9)

MarriottCharlotte F.^1^*BrierleyLaura-Beth^1^BrazierJohn A.^1^BovillRichard^2^OsbornHelen M. I.^1^1Reading School of Pharmacy, University of Reading, P.O. Box 226, Whiteknights, Reading RG6 6AP, UK2Thermofisher, Wade Road, Basingstoke, Hampshire RG24 8PW, UK*Correspondence: C.Marriott@pgr.reading.ac.uk

In recent years, there has been an alarming increase in bacteria that are resistant to existing antibiotics. In a review on antimicrobial resistance antimicrobial resistance was predicted to be the main cause of deaths globally in 2050, overtaking cancer (O’Neil, J. Tackling drug-resistant infections globally: final report and recommendations, Review 2016). These resistant bacteria or ‘superbugs’ also are also important financially and over £1 billion a year is spent on Healthcare Associated Infections (HCAI) in the National Health Service with Methicillin-Resistant *Staphylococcus aureus* (MRSA) and *Clostridium difficile* (*C. difficile*) accounting for up to 15% of these HCAIs (Mantle, S. https://www.england.nhs.uk/wp-content/uploads/2015/04/10-amr-lon-reducing-hcai.pdf). There is thus a need to design, synthesise and test new antibiotics to fight against the new resistant bacteria.

The aim of this study was to provide structure-activity relationship details on the bis-phenyls compounds, bromochlorophen, dichlorophen and hexachlorophene. These halogenated bis-phenyls were used in the past as biocides but they were withdrawn due to toxicity. Using a variety of techniques, glycosylated prodrugs of these compounds were synthesised and tested. Glycosylation reduced the toxicities of the compounds, with the more halogenated compounds still being the most toxic.

### 5.7. Design, Synthesis and Evaluation of Isatin-Based Estrogen Receptor Ligands (P10)

NooraniSara*ArfaeiSaraMeeganMary J.Trinity Biomedical Sciences Institute, 152-160 Pearse Street, Trinity College Dublin, Dublin 2, Ireland*Correspondence: noorani87@gmail.com

Breast cancer is classified into different subgroups by the expression of estrogen receptor (ER), progesterone receptor (PR) and human epidermal growth factor receptor 2 (HER2). These types of breast cancers present with distinct molecular backgrounds and exhibit diverse clinical behavior and response to treatments. Tumors with negative expression of ER, (25% to 30% of breast cancers), are characterised by their high metastatic potential and poor prognosis. The discovery of novel therapeutic treatments is required to advance the clinical outcomes for patients with ER-negative breast cancers. Tamoxifen is an endocrine therapy for the adjuvant treatment of Estrogen Receptor (ER) positive breast cancer. We have developed a novel series of benzoxepin-containing estrogen receptor modulators which bind to its receptor with a considerably higher potency than tamoxifen, while also inhibiting the proliferation of a human MCF-7 breast carcinoma cell line (O’Boyle, N.M., et al. *J. Med. Chem.* **2017**, *61*, doi:10.1021/acs.jmedchem.6b01917). The related novel heterocyclic structures are designed to incorporate the potent isatin scaffold structures. Compounds which modulate the estrogen receptors (ER), which act as either agonist or antagonists, or in a tissue selective manner are recognised for their pharmaceutical utility in the treatment of a wide variety of conditions related to the CNS, skeletal system, reproductive system, cardiovascular system, skin and immune system as well as estrogen receptor-and non-receptor expressing tumours. As a result, SERMs and SERDs offer the benefits associated with estrogens on bones (and on lipid levels in the blood), but do not appear to have the negative side effects associated with estrogens in breast and endometrial tissues.

The series of novel compounds synthesised have been characterized (^1^H-NMR, ^13^C-NMR, IR, HRMS). The antiproliferative activity has been evaluated on the human breast cancer MCF-7 cell line and the estrogen receptor-negative breast cancer MDA-MB-231 cell line. The IC_50_ values for the most active compounds were obtained at submicromolar levels. A library of isatin-based ER antagonists and related compounds has been successfully synthesised and the biological evaluation demonstrated promising results in human breast cancer cells. The pro-apoptotic effects of these compounds in breast cancer cell lines were shown to be significantly more potent than tamoxifen with Annexin V assay, and the compounds also displayed cytotoxic activity in a range of diverse human cancer cell lines. This study allows further lead optimization of this class of compounds as potential estrogen receptor-targeting agents.


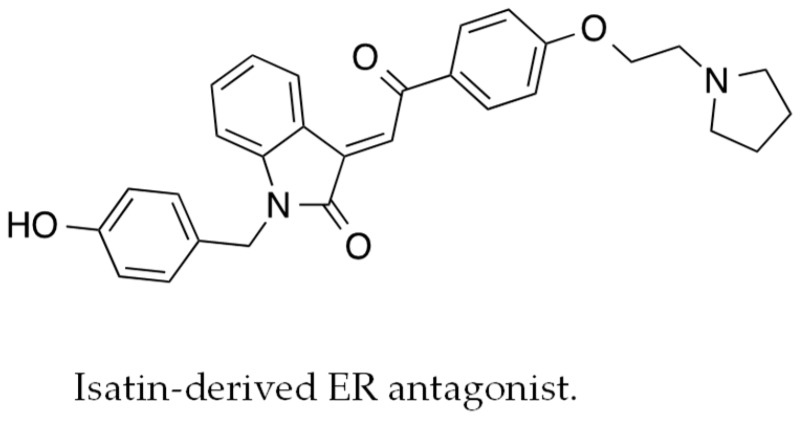


### 5.8. Enantiopure 2,4,7-Trisubstituted Fluorene Derivatives as Novel Antimalarial Agents (P12)

SchneiderJérémy*Dassonville-KlimptAlexandraSonnetPascalLG2A, CNRS UMR 7378, UFR de Pharmacie, Université de Picardie Jules Verne, 1 rue des Louvels, 80037 Amiens CEDEX, France*Correspondence: jeremy.schneider@etud.u-picardie.fr

Malaria is a neglected tropical disease that remains a leading cause of morbidity and mortality among the world’s poorest populations. More than 100 tropical and sub-tropical countries are endemic for this infectious disease. Pregnant women and children are the most sensitive to this infection and, in 2015, 429,000 people died (World Malaria Report, WHO, 2016, 2–8). Among the five species of *Plasmodium* responsible for human malaria, *P. falciparum* is the parasite which causes the most serious form of the disease. More recent efforts focused on the development of antimalarial vaccines and since 2001, World Health Organization (WHO) recommends artemisinin-based combination therapies (ACTs) (Guidelines for the treatment of malaria, 3rd ed., WHO, 2015; pp. 214–219; Jonet, A., et al. *Tetrahedron Asymm*. **2011**, *22*, 138–148). In drugs resistance areas, several antimalarial drugs, such as aminoaryl-alcohol (mefloquine (MQ), lumefantrine (LM)), are currently used in combination with artemisinin derivatives. However, the emergence of multi-drug-resistant parasites decreases efficacy of ACTs. Thus, the design of new active compounds on *Plasmodium*-resistant strains is urgently needed.

We have previously developed an asymmetric synthesis to prepare 4-aminoquinoline-methanol enantiomers (AQM) as MQ analogs. They were active on nanomolar range against 3D7 (chloroquine-sensitive) and W2 (chloroquine-resistant) *P. falciparum* strains. Interestingly, (*S*)-enantiomers displayed an activity increased by 2 to 15-fold as compared to their (*R*)-counterparts. During the *Plasmodium* intra-erythrocytic asexual stages, hemozoin formation and the oxidative and glutathione-dependent degradation of heme are inhibited by these amino-aryl-alcohols (MQ, LM). Currently their mechanisms of actions are not totally clear and remain to be explored (Mullié, C., et al. *Malaria J*. **2012**, *11*, 65; Dassonville-Klimpt, A., et al. Microbiology Book Series—2011; Méndez-Vilas, A., Ed.; 2011; Petersen, I., et al. *FEBS Lett.* **2011**, *585*, 1551–1562).

In continuation of our work, we are interested now to study the change of heterocycle (fluorene vs. quinoline) on the antimalarial activity. We focus on the design and the preparation of novel asymmetric 2,4,7-trisusbtituted fluorenes, new aminofluorene-methanol derivatives (AFM) as LM analogs. The evaluation of their antiplasmodial activity against *P. falciparum* and their corresponding cytotoxicity is under progress.

**Acknowledgments:** J.S. was the recipient of a grant from DGA (Direction Générale de l’Armement, Ministère de la Défense, France) and Région Picardie.

### 5.9. Synthesis and Biological Evaluation of Novel 5-HT_6_ Receptor Antagonists (P13)

ToubletFrançois-Xavier*LalutJulienLecouteyCédricDavisAudreyRochaisChristopheDallemagnePatrickCentre d’Etudes et de Recherche sur le Médicament de Normandie (CERMN)-UPRES EA 4258-FR CNRS INC3M-SFICORE, Université de Caen Normandie, UFR Santé-Bd Becquerel, F-14032 Caen, France*Correspondence: francois-xavier.toublet@unicaen.fr

Alzheimer’s disease is a multifactorial neurodegenerative disease still poorly understood. The molecular origin of the disease is the formation of amyloid plaques, caused by a hyperactivation of β-secretase forming the β-amyloïd peptide, and the hyperphosphorylation of tau protein, leading to neurofibrillary tangles and disaggregation of microtubules. Because of the molecular complexity of the disease, an impressive number of therapeutic targets have been envisaged. However, the market counts few molecules with moderate activity, mostly acetylcholinesterase inhibitors.

Among those targets the modulation of 5-HT receptor is of particular interest with the development of several antagonists in the clinical trials (Yun, H., et al. *Exp. Neurobiol*. **2011**, *20*, 159–168). Many studies prove that 5-HT_6_R antagonist increase cholinergic and glutamatergic neurotransmission, reversing memory disorders (Quiedeville, A., et al. *Behav. Brain Res*. **2015**, *293*, 10–17). Actually, a promising molecule, idalopirdine, is in phase III of the clinical trials, with patients in moderate stage diseases (Wilkinson, D., et al. *Lancet Neurol.* **2014**, *13*, 1092–1099). This antagonist allowed in the phase II, a significate amelioration after 24 weeks of treatments (Galimberti, D., et al. *Expert Opin. Investig. Drugs* **2015**, *24*, 1–7).

This molecule differentiates from other antagonists by an unusual structure, different from theoretical pharmacophore.

This project will present our effort toward the synthesis and in vitro evaluation of novel idalopirdine analogs.

### 5.10. Extraction and Purification of δ-Tocotrienol from Bixa orellana and Semi-Synthesis of 7-Formyl-δ-tocotrienol (P14)

AlsabilK.^1^ChererS. Suor^1^LamH. Y.^1^ViaultGuillaume^1^*HélesbeuxJean-Jacques^1^Peña-RodriguezL. M.^2^RichommePascal^1^SéraphinDenis^1^1Laboratoire SONAS EA921, SFR QUASAV, Université d’Angers, 49035 Angers, France2Grupo de Química Orgánica, Unidad de Biotecnología, Mérida, Yucatán 97440, Mexico*Correspondence: guillaume.viault@univ-angers.fr

Vitamin E was first discovered in 1922 and is well known for its anti-oxidant activities. Actually, Vitamin E is a group of 8 isoforms classified into α-, β-, γ- and δ-tocopherols and α-, β-, γ- and δ-tocotrienols. Changes of the structures are the degree of methylation of the chromanol ring and the saturation of the side chain. Nevertheless, vitamin E has been associated with tocopherols over decades. Biological activities studies tended to focus on that series but recently, it was found that tocotrienols were of greater pharmacological potentials (Ahsan, H., et al. *Nutr. Metab*. **2014**, *11*, 52). They possess prevailing neuroprotective, anti-cancer, anti-inflammatory and cholesterollowering properties. Therefore, tocotrienols and derivatives are one of our major topics of interest.

Two formylated-δ-tocotrienols, namely 5-formyl-δ-tocotrienol and 7-formyl-δ-tocotrienol, have been isolated from the stem bark of *Garcinia virgata* (Merza, J., et al. *Phytochemistry* **2004**, *65*, 2915–2920), and could be used to introduce further structural diversity. However, the insufficient amount isolated did not allow the full spectroscopic characterization of 7-formyl-δ-tocotrienol. Purification of a dichloromethane extract of *Bixa orellana* seeds provided pure δ-tocotrienol and allowed us to semisynthesize the 7-formyl-δ-tocotrienol in three steps.

### 5.11. Semisynthetics Pathways Starting from δ-Garcinoic Acid towards Anti-Inflammatory Tocotrienols Isolated from New-Caledonian Endemic Plants (P15)

VilleAlexia*ViaultGuillaumeHélesbeuxJean-JacquesSéraphinDenisSONAS EA921, SFR QUASAV 4207, Université d’Angers, 42 Rue Georges Morel, 49070 Beaucouzé, France*Correspondence: alexia.ville@univ-angers.fr

Vitamin E (VE) includes eight chemically distinct chromanols named α-, β-, γ-, δ-tocopherols (T) and tocotrienols (T3). They mainly differ by the number and the position of aromatic methyl groups and by the nature of the side chain, phytyl for T and farnesyl for T3 (Theriault, A., et al. *Clin. Biochem.* **1999**, *32*, 309–319). Besides VE, analogs oxidized on the terminal methyl groups of the side chain (e.g., carboxylic acid for garcinoic acid, GA or diol for amplexichromanol, AC) were also isolated from vegetal sources. Amongst the various bioactivities reported for this class of compounds, T3 derivatives showed hypocholesterolemic, anti-cancer and neuroprotective propertiesa. More specifically, δ-AC has shown significant antiangiogenic activity (IC_50_ = 25 nM) (Lavaud, A., et al. *J. Nat. Prod.* **2013**, *76*, 2246–2252).

Tocotrienolic secondary metabolites (T3) are found in several plants mainly growing in tropical areas (Lavaud, A. Doctoral thesis 2012ANGE0051, Université d’Angers, Beaucouzé, France, 2012). However, δ-T3 purification from easily available plant materials (*Bixa orellana*, *Hevea brasiliensis*) is tedious because of the presence of all the VE isoforms. Previously we identified two plants with a high δ-T3 content (*Mammea neurophylla*, 0.5% (Dang, B.T., et al. *Fitoterapia* **2014**, *96*, 65–75)) or δ-AC content (Garcinia amplexicaulis, 1.6% (Lavaud A. Doctoral thesis 2012ANGE0051, Université d’Angers, Beaucouzé, France, 2012)) that are endemic from New Caledonia and thus difficult to obtain. Moreover, both metabolites are isolated from the barks of these trees, as non-renewable vegetal materials (Lavaud A. Doctoral thesis 2012ANGE0051, Université d’Angers, Beaucouzé, France, 2012; Dang, B.T., et al. *Fitoterapia* **2014**, *96*, 65–75).

*Garcinia kola* is a tree, largely distributed in African countries, whose seeds are used in traditional medecine (Terashima, K., et al. *Bioorg. Med. Chem*. **2002**, *10*, 1619–1625). Large amounts (1%) of δ-GA were isolated from this renewable material even available in ethnic groceries in France. Thus, we thought δ-GA could be a convenient platform to access to δ-T3 and δ-AC through semisynthetic strategies.

### 5.12. Building a Diverse and Experimentally-Curated Fragment Library (P17)

BroughSteven^1^*LowersonAndrew^1^LaPlanteSteven^2^McCarrenPatrick^3^Serrano-WuMichael^3^1Key Organics Limited, Cornwall PL32 9RA, UK2NMX Research and Solutions, Montreal, QC H7V 5B7, Canada3Broad Institute, Cambridge, MA 02142, USA*Correspondence: steveb@keyorganics.net

Fragment libraries are commonly assembled by Rule of 3 filtering followed by manual curation. However, the robust experimental data that ensures the proper physicochemical attributes needed for high-concentration screening is often lacking and replaced instead by in silico calculations of uncertain predictive value. A fragment collection with experimentally-determined aqueous solubility will address a major source of false positives and attrition in fragment screening libraries: Aggregation, Stability, and Solubility. ^1^H-NMR spectral data in aqueous buffer will further enable practitioners to rapidly build fragment pools and initiate screening.

Diversity selection methods in shape, scaffold, fingerprint, and predicted property space combined with industry-standard substructure filtering were used to select over 2500 Key Organics compounds for experimental profiling. NMR and LCMS analysis allowed the careful selection of highly-soluble fragments with desirable physicochemical and stability characteristics. Importantly, the curated molecules are enriched in cyclic scaffolds commonly found in drug candidates, and spans chemical space that minimally overlaps with existing commercial collections. This poster will summarize the experimental and cheminformatic features of this next-generation Key Organics ‘BIONET Premium Fragment Library’ (Congreve, M., et al. *Drug Discov. Today* **2003**, *8*, 876–877; Hall, R.J., et al. *Prog. Biophys. Mol. Biol*. **2014**, *116*, 82–91; Pipeline Pilot v 8.5; note H-bond acceptors and donors used the default Accelrys Num_H_Acceptors and Num_H_Donors which are different from the Lipinski definition; Bruns, R.F., et al. *J. Med. Chem*. **2012**, *55*, 9763–9772; Baell, J.B., et al. *J. Med. Chem.* **2010**, *53*, 2719–2740; Pearce, B.C., et al. *J. Chem. Inf. Model*. **2006**, *46*, 1060–1068; Kombarov, R., et al. *Mol. Divers.* **2010**, *14*, 193–200; Conformations generated in MOE 2014 using the MMFFs force field; Bemis-Murcko scaffolds were calculated as in the defaults for the Pipeline Pilot Generate Fragments, which keeps atom type; Akoka, S., et al. *Anal. Chem*. **1999**, *71*, 2554–2557).

### 5.13. Synthesis and Biochemical Evaluation of Novel Heterocyclic Stilbene Compounds with Tubulin Targeting Activity (P18)

AnaGloriaO’BoyleNiamhMeeganMary J.*Trinity Biomedical Sciences Institute, 152-160 Pearse Street, Trinity College Dublin, Dublin 2, Ireland*Correspondence: mmeegan@tcd.ie

Drug-like small molecule natural product such as colchicine and the combretastatins provide ideal structural templates for the design of structural scaffolds with improved efficacy as tubulin targeting agents. Combretastatin A-4 originally isolated from Combretum caffrum, exhibits strong antitubulin activity by binding to the colchicine-binding site (Pettit G., et al. *J. Med. Chem*. **1998**, *41*, 1688–1695) with highly potent cyctotoxicity against a variety of human cancers including multidrug resistant (MDR) cancer cell lines it also inhibits angiogenesis, an essential process for the tumor growth (Provot, O., et al. *Anti-Cancer Agents Med. Chem*. **2013**, *13*, 1614–1635). In this work, a library of amide conjugates of combretastatin A-4 and related structures have been synthesized and the biological activity has been investigated. We report our synthetic approaches to stilbene analogues of colchicine, combretastatin-A4 and phenstatin together with their in vitro biological activity. We also report molecular docking studies and the SAR of these compound classes as tubulin targeting agents, which have underlined that the presence of the 3,4,5 trimethoxy substituted A-ring and the 4-methoxy substituted B-ring separated by a double-bond to be essential for antiproliferative activity. The activity of CA4 is limited by isomerization of the active *cis*-stibene configuration into the inactive *trans* analogue. Introduction of a heterocyclic amide group on the stilbene scaffold may create a stabilised CA4-like compound locked in its *cis*-form.

The first step in the synthesis of our library of stilbene derivatives is the Perkin reaction of an aromatic aldehyde and a substituted phenylacetic acid, to afford the acrylic acid combretastatin analogues. The reaction has now been optimized using a microwave reactor. The newly synthesised acrylic acids have then been coupled to a variety of heterocyclic amides using the Mukaiyama reagent as coupling reagent (including appropriate protecting/deprotecting steps) to afford the heterocyclic amide conjugates with piperlongumine type structures (Meegan, M.J., et al. *Eur. J. Med. Chem.* **2017**, *125*, 453–463). The novel compounds synthesised have been characterised (^1^H-NMR, ^13^C-NMR, IR, HRMS). The purity of the final products has been evaluated by HPLC and when possible the structure of the compounds was established by single-crystal X-ray analysis. The biological activity has been evaluated on the human MCF-7 cell line in an antiproliferative assay and the IC_50_ value for the most active compounds obtained. A library of heterocyclic-combretastatin conjugates and related compounds has been successfully synthesised and the biological evaluation demonstrated promising results in human breast cancer cells. This study allows further lead optimisation of this class of compounds as potential tubulin-targeting compounds.

### 5.14. Synthesis of Teixobactin and Analogues (P21)

ThomasCarys*GanesanA.School of Pharmacy, University of East Anglia, Norwich NR7 4TJ, UK*Correspondence: Carys.Thomas@uea.ac.uk

The emergence of antibiotic-resistant strains of bacteria has led to an urgent need for new antibiotics, since antibiotic resistance is now one of the major challenges faced by medicine. A possible solution to this problem is to investigate new natural products showing promising antibacterial activity. Therefore, the reporting of the discovery of teixobactin in 2015, showing good activity against Gram-positive bacteria, including drug-resistant strains, generated much interest (Ling, L.L., et al. *Nature* **2015**, *517*, 455–459).

To develop the potential of this drug candidate further, synthesis and understanding of the structure- activity relationships of this new natural product is essential. Previous research (Yang, H., et al. *ACS Chem. Biol.* **2016**, *11*, 1823−1826) has found that the structure of the macrocycle is important for good activity but activity can be retained with modifications to the linear tail. Analogues can be synthesised by firstly using solution-phase synthesis to make large quantities of the macrocycle, initially substituting arginine for the rare non-proteinogenic amino-acid enduracididine. Solid-phase synthesis is used to form heptapeptides to couple to the macrocycle to make a series of analogues, which can then be tested for activity. 

Synthesis of enduracididine has also been carried out, allowing total synthesis using the same method as for analogues (Figure 1).

### 5.15. Probing Imidazotetrazine Prodrug Activation Mechanisms (P22)

MoodyCatherine L.AhmadLeenaAshourAhmedWheelhouseRichard T.*School of Pharmacy and Medical Sciences, University of Bradford, Bradford BD7 1DP, UK*Correspondence: r.t.wheelhouse@bradford.ac.uk

The archetypal prodrug of the imidazotetrazine class is the anticancer agent temozolomide (TMZ). The prodrug activation kinetics of TMZ show an unusual pH dependence: it is stable in acid and rapidly hydrolyses in alkali (Denny, B.J., et al. *Biochemistry* **1994**, *33*, 9045–9051). The incipient drug MTIC has the opposite properties—relatively stable in alkali but unstable in acid. In this study, the mechanism of prodrug activation was probed in greater detail to determine whether the reactions are specific or general acid or base catalysed. Three prodrugs and drugs were investigated, TMZ, MTIC and the novel dimeric imidazotetrazine EA27. Hydrolysis in a range of citrate-phosphate buffers (pH 8.0, 7.4, 4.0) was measured by UV spectrophotometry. 

Reaction of TMZ and MTIC obeyed single-phase, pseudo-first order kinetics (Figure 1). EA27 was more complex, showing biphasic but approximately pseudo-first order kinetics, Figure. General acid or base catalysis indicates that protonation or deprotonation is the rate-limiting step (rls). In biological milieu, the nature and concentration of other acidic or basic solutes may affect the prodrug activation reaction. In contrast, specific acid or base catalysis indicates that protonation or deprotonation occurs before the rls, so catalysis depends only on the local concentration of hydroxide or hydronium ion (i.e., pH) so the reaction kinetics are not expected to change appreciably in a biological medium.

The difference in reactivity between the tetrazines of the symmetrical dimer EA27 is surprising. This suggests a sequential mechanism where the hydrolysis of one imidazotetrazine slows the rate of the second within the same molecule, even though the two are FIVE atoms apart and not conjugated. The switch of mechanism (especially at pH 8) between TMZ and EA27 implies a role for the intramolecular secondary amine at the rate-limiting step.

**Acknowledgments:** This work was supported in part by Yorkshire Cancer Research.

### 5.16. Mechanisms of Action of Silane-Substituted Anti-Cancer Imidazotetrazines (P23)

SummersHelen S.^1^BradshawTracey D.^1^StevensMalcolm F. G.^1^WheelhouseRichard T.^2^*1School of Pharmacy, University of Nottingham, Nottingham NG7 2RD, UK2School of Pharmacy and Medical Sciences, University of Bradford, Bradford BD7 1DP, UK*Correspondence: r.t.wheelhouse@bradford.ac.uk

Silane-substituted imidazotetrazines **1**,**2** were investigated for their activity as anticancer prodrugs related to temozolomide (TMZ). The TMS-derivative **1** showed an activity profile against TMZ susceptible and resistant cell lines very similar to TMZ; in contrast, the SEM-derivative **2** showed activity irrespective of MGMT expression or MMR deficiency (Table).

Probing the prodrug activation mechanism by NMR kinetic studies determined that the TMS compound **1** follows a reaction pathway and time-course very similar to temozolomide. ^1^H-NMR spectra of the reaction mixture showed considerable incorporation of deuterium into the final alkylation products of the reaction (methanol and methyl phosphate) as had previously been shown for temozolomide (Wheelhouse, R.T., et al. *Chem. Commun*. **1993**, *15*, 1177–1178). The SEM-derivative **2** reacted more rapidly than TMZ or TMS-derivative **1**. Somewhat surprisingly, the silane remained intact throughout the experiment and the observed reaction was the hydrolysis of the imidazo-tetrazine to ultimately release formaldehyde hydrate and 2-TMS-ethanol.


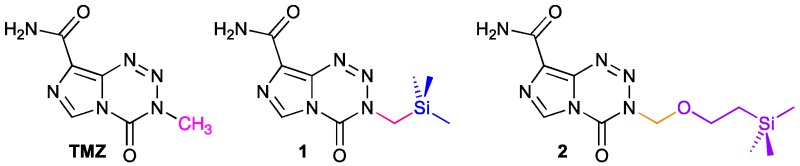


Activity of TMZ, compounds **1** and **2** against a panel of TMZ susceptible and resistant cell lines**Compound****GI_50_ Value ± SD (µM)****U373 V****U373 M****HCT 116****TMZ**51.9 ± 7.4302 ± 56291 ± 4.9**1**61.0 ± 6.0>500233 ± 80**2**36.1 ± 2.929.1 ± 2.532.3 ± 10

In conclusion, TMS-derivative **1** is a diazomethane precursor with prodrug activation mechanism, kinetics and anti-cancer activity in vitro similar to TMZ. In contrast, the SEM derivative **2** was more rapidly hydrolysed, a precursor of 2-TMS-ethanol and had activity in vitro different from TMZ. 2-TMS-ethanol was previously reported as a non-toxic compound in mice (Voronkov, M.G., et al. *Dokl. Akad. Nauk SSSR* **1976**, *229*, 1011–1013) and is known as a substrate for alcohol dehydrogenase (Zong, M.-H., et al. *Appl. Microbiol. Biotechnol*. **1991**, *36*, 40–43) and as a modest inhibitor of acetylcholinesterase (Aberman, A., et al. *Biochim. Biophys. Acta* **1984**, *791*, 278–280; Cohen, S.G., et al. *J. Med. Chem*. **1985**, *28*, 1309–1313).

## 6. Conclusions

The meeting was a success in its primary aims of bringing young researchers together to exchange scientific ideas and experiences. New collaborations, formal and informal, were forged. Reflecting the high standard of oral and poster presentations, prizes were awarded to young medicinal chemists. Dr. Amit Nathubhai (University of Bath, Bath, UK) received the award for the best submitted oral presentation, Isabelle Lengers (University of Münster, Münster, Germany) for the best poster with flash oral presentation (sponsored by the Society of Chemical Industry Young Chemists Panel, London, UK). Mike Kenny (University of Bath, Bath, UK) and Alexia Ville (Université d’Angers, Angers, France) received awards for best posters (Sponsored by the Royal Society of Chemistry Liverpool Local Section, Liverpool, UK).

The 26th Annual Meeting of GP2A is scheduled for 13–15 June 2018 in Asnelles-sur-Mer (Normandie, France), as a joint meeting with the 32nd Journées Franco-Belges de Pharmacochimie.

With the election of a new President and new Committee for GP2A, the organisation looks forward to developing and expanding its activities, fostering greater collaborations between member laboratories and expanding into more countries in Western Europe.

## Figures and Tables

**Figure 1:**
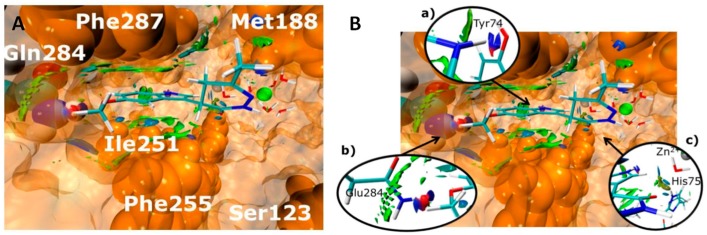
PDE4-3c interface, using promolecular densities. (**A**) best pose (⊗G = −5.3 kcal·mol^−1^) and (**B**) focus on specific interactions.

**Figure 1:**
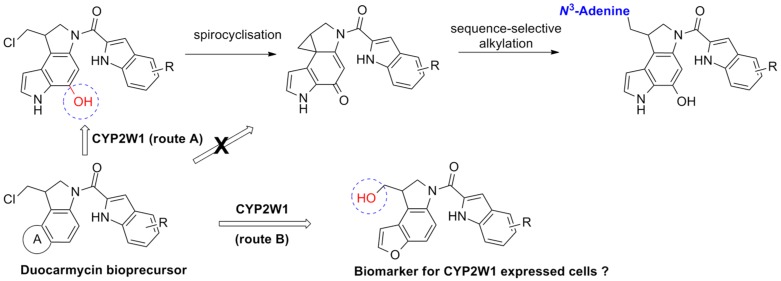
Proposed pathway for CYP activation of duocarmycin biopersursor substrates^3,4^ (A = pyrrole or furan ring).

**Figure 1:**
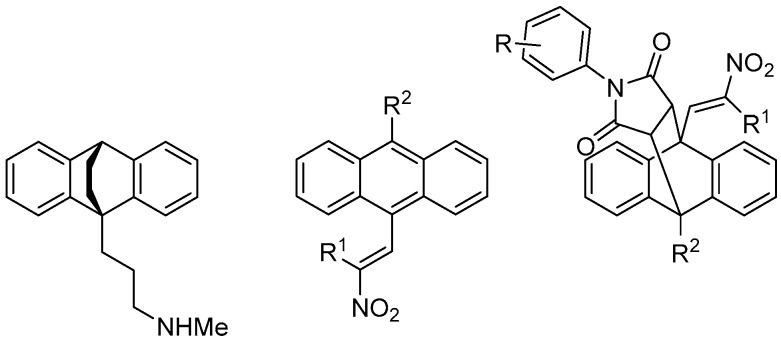
Maprotiline, (*E*)-9-(2-nitrovinyl)anthracenes and ethanoanthracene derivatives.

**Figure 1:**
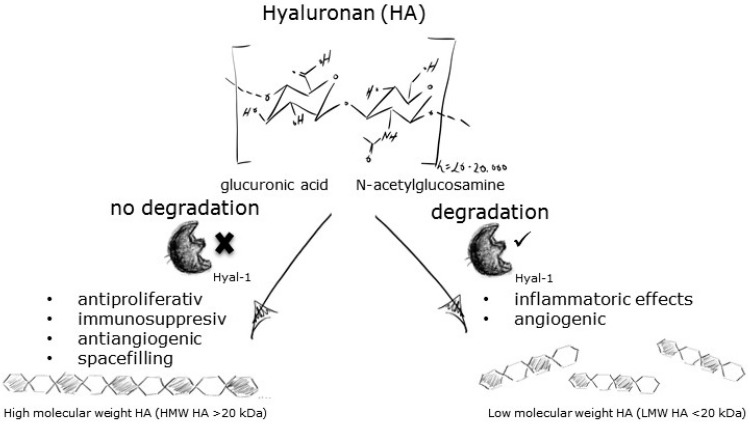
Physiological and pathophysiological functions of hyaluronan.

**Figure 1:**
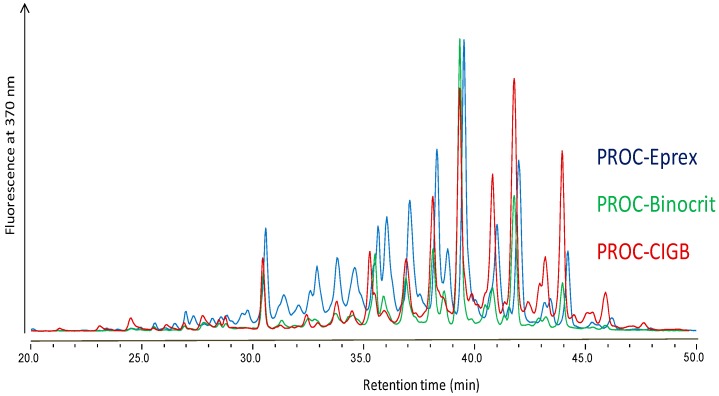
Liquid chromatography (LC) analysis of procainamide (PROC) labelled *N*-glycans contained in Eprex, Binocrit and CIGB-EPO.

**Figure 1:**
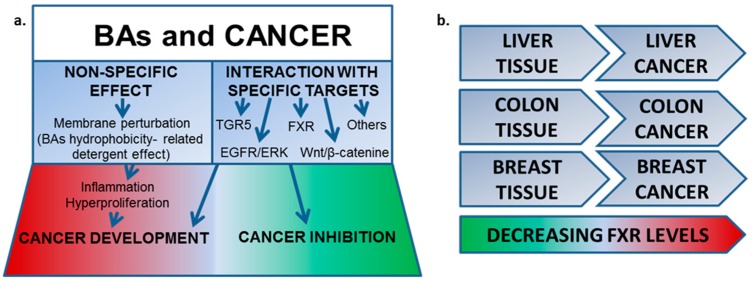
(**a**) Schematic summary of the involvement of BAs in cancer; (**b**) FXR expression decreases from healthy to cancer tissues.

**Figure 1:**
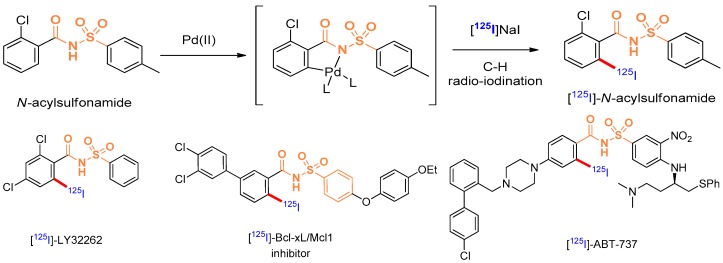
(**Upper**) Palladium-mediated C-H radioiodination. (**Lower**) Structures of [125I]-LY32262, a [125I]-Bcl-xL/Mcl1 and [125I]-ABT-737.

**Figure 1:**
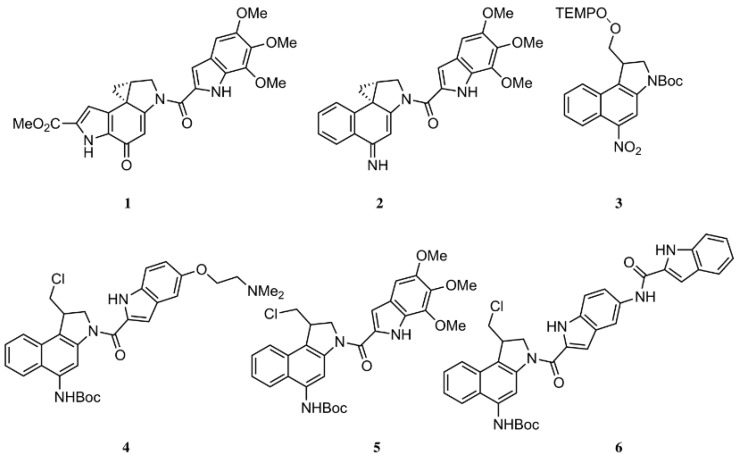
Structures of Duocarmycin SA, **1**, amino-CBI, **2**, Key intermediate, **3**, final *seco*-amino-CBIs, **4**–**6**.

**Figure 1:**
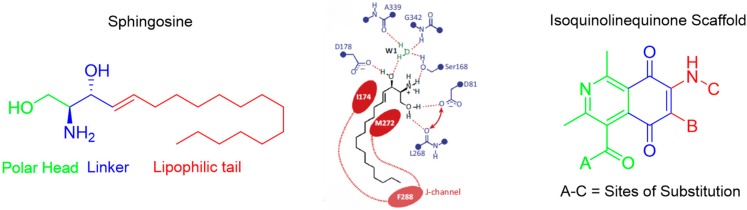
Sphingosine docking into the narrow J-channel in the active site of SK1. Transposing the general regions of sphingosine onto the proposed isoquinolinequinone template affords a versatile, novel potential inhibitor.

**Figure 1:**
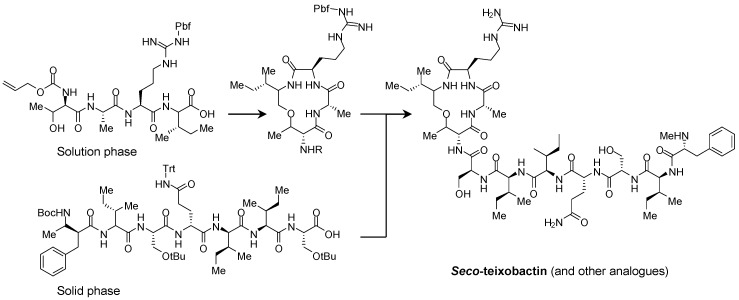
Outline of the synthesis of teixobactin analogues.

**Figure 1:**
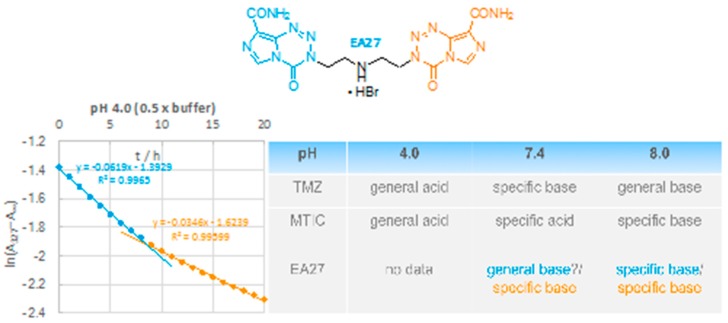
EA27 (top), example hydrolysis data (L) and the prodrug activation mechanisms determined (R).

